# Effect of Disposable Elevator Cap Duodenoscopes on Persistent Microbial Contamination and Technical Performance of Endoscopic Retrograde Cholangiopancreatography: The ICECAP Randomized Clinical Trial

**DOI:** 10.1001/jamainternmed.2022.6394

**Published:** 2023-01-23

**Authors:** Nauzer Forbes, B. Joseph Elmunzer, Thibault Allain, Michael D. Parkins, Prameet M. Sheth, Barbara J. Waddell, Kristine Du, Katya Douchant, Olajumoke Oladipo, April Saleem, Shane Cartwright, Millie Chau, Megan Howarth, Jackie McKay, Tamim Nashad, Yibing Ruan, Kirles Bishay, Emmanuel Gonzalez-Moreno, Zhao Wu Meng, Sydney Bass, Robert Bechara, Martin J. Cole, Diederick W. Jalink, Rachid Mohamed, Christian Turbide, Paul J. Belletrutti, Ahmed Kayal, Puja R. Kumar, Robert J. Hilsden, André G. Buret, Lawrence Hookey, Steven J. Heitman

**Affiliations:** 1 Division of Gastroenterology and Hepatology, Department of Medicine, University of Calgary, Calgary, Alberta, Canada; 2 Department of Community Health Sciences, University of Calgary, Calgary, Alberta, Canada; 3 Division of Gastroenterology and Hepatology, Medical University of South Carolina, Charleston; 4 Department of Biological Science, University of Calgary, Calgary, Alberta, Canada; 5 Department of Microbiology, Immunology and Infectious Diseases, University of Calgary, Calgary, Alberta, Canada; 6 Division of Infectious Diseases, Department of Medicine, University of Calgary, Calgary, Alberta, Canada; 7 Department of Biomedical and Molecular Sciences, Queen’s University, Kingston, Ontario, Canada; 8 Department of Pathology and Molecular Medicine, Queen’s University, Kingston, Ontario, Canada; 9 Department of Translational Medicine, Queen’s University, Kingston, Ontario, Canada; 10 Department of Oncology, Cumming School of Medicine, University of Calgary, Calgary, Alberta, Canada; 11 Department of Cancer Epidemiology and Prevention Research, Cancer Care Alberta, Alberta Health Services, Calgary, Alberta, Canada; 12 Division of Gastroenterology, Department of Medicine, Queen’s University, Kingston, Ontario, Canada; 13 Department of Surgery, Queen’s University, Kingston, Ontario, Canada

## Abstract

**Importance:**

Infection transmission following endoscopic retrograde cholangiopancreatography (ERCP) can occur due to persistent contamination of duodenoscopes despite high-level disinfection to completely eliminate microorganisms on the instrument.

**Objective:**

To determine (1) contamination rates after high-level disinfection and (2) technical performance of duodenoscopes with disposable elevator caps compared with those with standard designs.

**Design, Setting, and Participants:**

In this parallel-arm multicenter randomized clinical trial at 2 tertiary ERCP centers in Canada, all patients 18 years and older and undergoing ERCP for any indication were eligible.

**Intervention:**

The intervention was use of duodenoscopes with disposable elevator caps compared with duodenoscopes with a standard design.

**Main Outcomes and Measures:**

Coprimary outcomes were persistent microbial contamination of the duodenoscope elevator or channel, defined as growth of at least 10 colony-forming units of any organism or any growth of gram-negative bacteria following high-level disinfection (superiority outcome), and technical success of ERCP according to a priori criteria (noninferiority outcome with an a priori noninferiority margin of 7%), assessed by blinded reviewers.

**Results:**

From December 2019 to February 2022, 518 patients were enrolled (259 disposable elevator cap duodenoscopes, 259 standard duodenoscopes). Patients had a mean (SD) age of 60.7 (17.0) years and 258 (49.8%) were female. No significant differences were observed between study groups, including in ERCP difficulty. Persistent microbial contamination was detected in 11.2% (24 of 214) of standard duodenoscopes and 3.8% (8 of 208) of disposable elevator cap duodenoscopes (*P* = .004), corresponding to a relative risk of 0.34 (95% CI, 0.16-0.75) and number needed to treat of 13.6 (95% CI, 8.1-42.7) to avoid persistent contamination. Technical success using the disposable cap scope was noninferior to that of the standard scope (94.6% vs 90.7%, *P* = .13). There were no differences between study groups in adverse events and other secondary outcomes.

**Conclusions and Relevance:**

In this randomized clinical trial, disposable elevator cap duodenoscopes exhibited reduced contamination following high-level disinfection compared with standard scope designs, without affecting the technical performance and safety of ERCP.

**Trial Registration:**

ClinicalTrials.gov Identifier: NCT04040504

## Key Points

**Question:**

In patients undergoing endoscopic retrograde cholangiopancreatography (ERCP), do duodenoscopes with disposable elevator caps (1) reduce contamination rates after high-level disinfection and/or (2) maintain technical performance of the procedure compared with duodenoscopes with standard designs?

**Findings:**

In this randomized clinical trial that included 518 patients undergoing ERCP of various procedural complexity, duodenoscopes with disposable caps reduced persistent microbial contamination (relative risk, 0.34), with no differences in performance (technical success, 94.6% vs 90.7%) and safety outcomes.

**Meaning:**

Disposable elevator cap duodenoscopes exhibited reduced contamination following high-level disinfection compared with standard scope designs, without affecting technical performance and safety of ERCP.

## Teaser

This randomized clinical trial determines contamination rates after high-level disinfection and technical performance of duodenoscopes with disposable elevator caps compared with those with standard designs.

## Introduction

Endoscopic retrograde cholangiopancreatography (ERCP) is the reference standard for management of pancreaticobiliary pathology.^1,2,3,4^ The ERCP procedure is performed using duodenoscopes—specialized endoscopes with elevator mechanisms—to assist with pancreaticobiliary access. Given their design, duodenoscopes are among the most complex devices requiring high-level disinfection. High-level disinfection uses chemical agents to completely eliminate all microorganisms on the duodenoscope, except for small numbers of bacterial spores.

There has been a sharp increase in global reporting of duodenoscope-related infectious outbreaks,^5,6,7,8^ with the majority unrelated to identifiable breaches in disinfection protocols.^9,10,11^ This has led experts to question the design of the standard duodenoscope. In the Netherlands, a recent nationwide study found that 22% of duodenoscopes were persistently contaminated after high-level disinfection.^12^ As the mortality associated with duodenoscope-related sepsis can approach 29%,^6^ several organizations have declared this issue a priority,^13^ with the US Food and Drug Administration (FDA) issuing a recommendation to transition to duodenoscopes with innovative designs.^14^

Several novel duodenoscope designs have emerged, including entirely disposable duodenoscopes, whose high costs and suboptimal technical performance collectively limit widespread adoption in their current form.^15,16^ In contrast, duodenoscopes with disposable elevator caps (eFigure 1 in Supplement 1) have been developed and represent a promising potential solution. However, the potential for reduced contamination of these novel duodenoscopes remains unproven, and their clinical performance has only been assessed in an unblinded case series.^17^ In this randomized clinical trial, we aimed to evaluate the persistent microbial contamination rates and technical performance of duodenoscopes with disposable elevator caps compared with those with standard designs.

## Methods

### Study Design and Oversight

The Infection Control in ERCP using a Duodenoscope with a Disposable Cap (ICECAP) Trial was a blinded parallel-group randomized clinical trial conducted at 2 tertiary care centers in Canada that perform approximately 1600 and 500 ERCPs annually (Calgary, Alberta, and Kingston, Ontario). The trial was carried out in accordance with the Consolidated Standards of Reporting Trials (CONSORT) reporting guideline (eTable 1 in Supplement 1).^18^ A steering committee designed and supervised the trial, with Data Safety and Monitoring Board meetings held throughout the trial’s execution. Protocols (eTable 2 in Supplement 1) were approved by all participating institutional review boards and were published^19^ and registered (ClinicalTrials.gov: NCT04040504) (Supplement 2). Patients provided written informed consent. The study was funded by the American Society for Gastrointestinal Endoscopy (ASGE) and the Canadian Institutes of Health Research. Unrestricted temporary use of duodenoscopes was provided by Pentax Medical with no accompanying financial support. None of the above parties were involved in study conception, design, or execution, or in the interpretation and/or reporting of results.

### Patients

Patients aged 18 years and older undergoing ERCP for any indication were eligible. Exclusion criteria included unwillingness or inability to provide informed consent, contraindications to ERCP, pregnant or breastfeeding status, or predicted inability to complete a 30-day follow-up. A full list of eligibility criteria is provided in the protocol (eMethods 1 in Supplement 1).^19^

### Randomization and Intervention

Enrollment and data collection were performed using a secure electronic system (REDCap, Vanderbilt University), and randomization was performed using a secure electronic platform (randomize.net, Interrand Inc), which was also used for allocation concealment from patients, outcome assessors, and analysts. Patients were randomly assigned in a 1:1 ratio to undergo ERCP using a disposable elevator cap duodenoscope (ED34-i10T2, Pentax Medical) or a standard duodenoscope (ED34-i10T, Pentax Medical). Randomization occurred immediately preceding ERCP and was performed in blocks of 8, stratified by study site.

### Outcomes and Follow-up

There were 2 coprimary outcomes. The first was persistent microbial contamination following high-level disinfection, defined as either (1) growth of 10 or more colony-forming units (CFUs) of any organism, or (2) any growth of gram-negative bacteria, within 72 hours of plating. The second was the technical success of ERCP, determined independently by 2 outcome adjudicators blinded to group assignment based on a priori definitions (eTable 3 in Supplement 1). Disagreements were resolved by a third blinded adjudicator.

Secondary outcomes included mortality, patient tolerability,^20^ and adverse events within 30 days of ERCP, as defined by the ASGE Lexicon^21^ and with only events deemed *definitely* or *probably* related to ERCP being included.^22^ Adverse events included cholangitis, pancreatitis, bleeding, perforation, and cardiopulmonary adverse events.

### Sample Collection

The duodenoscopes in both study arms were required to have been in clinical use for greater than 12 but not more than 24 months. All duodenoscopes underwent 2 cycles of high-level disinfection prior to sample collection.^23^ Following disinfection and sterilization, all duodenoscopes were required to pass point-of-care adenosine triphosphate (ATP) scanning to assess for bioluminescence from microbial residue, with any failed scan resulting in the scope being sent for another disinfection cycle. Therefore, all duodenoscopes sampled in this study were those with a negative ATP scan result prior to sampling.

Microbiological sampling was performed on all duodenoscopes within 60 minutes of them being deemed cleared for clinical use by 2 trained staff members according to a protocol adapted from the FDA and the US Centers for Disease Control and Prevention^23^ (eMethods 2 in Supplement 1). Two samples were acquired from each duodenoscope: 1 from the elevator area (the elevator itself for standard duodenoscopes, and the cap attachment point for disposable elevator cap duodenoscopes) and 1 from the instrument channel (see eFigure 1 in Supplement 1). Samples were transported in sterile Dey-Engley neutralizing broth in coolers and plated within 6 hours of collection. Given limitations in laboratory bandwidth, transportation time, and plating time, a maximum of 3 patients per weekday could be enrolled (see eFigure 2 in Supplement 1).

### Microbiological Analysis

Samples were concentrated on 0.45-μm gridded membrane filters with vacuum manifold filtration. Filters were placed in Columbia blood agar (CBA) petri dishes, incubated at 37 °C in aerobic conditions, and checked for colonies at 24, 48, and 72 hours. Identified colonies were tested for growth on CBA, MacConkey agar, and Columbia nalidixic acid agar, and Gram staining identification was performed. Growth was reported in CFUs. Isolates were frozen brain heart infusion broth with 25% glycerol stocks at −80 °C. If (1) any growth was initially detected at at least 10 CFUs or (2) any gram-negative microorganism was detected, regardless of CFUs, those isolates were later replated and retrospectively identified by matrix-assisted laser desorption/ionization–time of flight (MALDI-TOF). Organisms that were not able to be identified by MALDI-TOF were flagged for attempted identification using 16S Sanger sequencing. The full microbiological analysis protocol is provided (eMethods 3 in Supplement 1).^24^

### Sample Size Calculations

The expected control persistent microbial contamination rate was 10%.^12,14^ We judged that a relative risk reduction of 70% with disposable elevator cap duodenoscopes would be well above a clinically meaningful benefit. Using Fisher exact test to test the 2-sided hypothesis that a disposable elevator cap duodenoscope is superior to a standard duodenoscope with a power of 80% and a 2-sided α of .05, we calculated that 426 patients would be required after accounting for a 10% possible error or loss rate. Our center’s data^25^ informed a baseline technical success rate of 92%. A noninferiority margin of 7% was chosen after considering the balance of outcomes and costs associated with colonization,^26^ infection,^27^ and technical failure.^25^ Using an unpooled *z*-test to test the 1-sided hypothesis that disposable elevator cap duodenoscopes are noninferior to standard duodenoscopes with a power of 80% and a 1-sided α of .025,^28^ we calculated that 520 patients would be required after accounting for 10% attrition. The first 96 patients were randomized for the purposes of assessing coprimary outcome 2 (technical success of ERCP) only, while the remaining patients had both outcomes assessed.

### Statistical Analyses

To compare proportions, 2-tailed Fisher exact tests were used where no more than 5 patients had a particular outcome in any group, whereas χ^2^ tests were used to compare proportions where each group had more than 5 patients. Outcomes were compared between groups in an intention-to-treat analysis without adjustment. Comparisons were reported using relative risks (RRs) and numbers needed to treat (NNTs) along with their respective 95% CIs. Post hoc subgroup analyses were performed according to sex (female or male), age (<65 or ≥65 years), study site (Calgary or Kingston), procedural complexity (I-II or III-IV),^29^ papilla status (native or prior sphincterotomy), disposition (inpatient or outpatient), biliary stent placement (vs none), and indication (suspected or confirmed malignant neoplasm or all others). In a sensitivity analysis, post hoc subgroup analyses were corrected for multiple comparisons using the conservative Bonferroni correction to construct 99% CIs to replace 95% CIs. All statistical analyses were performed using RStudio statistical software, version 1.2.1335 (R Project for Statistical Computing).

## Results

### Patient and Procedure Characteristics

**Figure 1.**
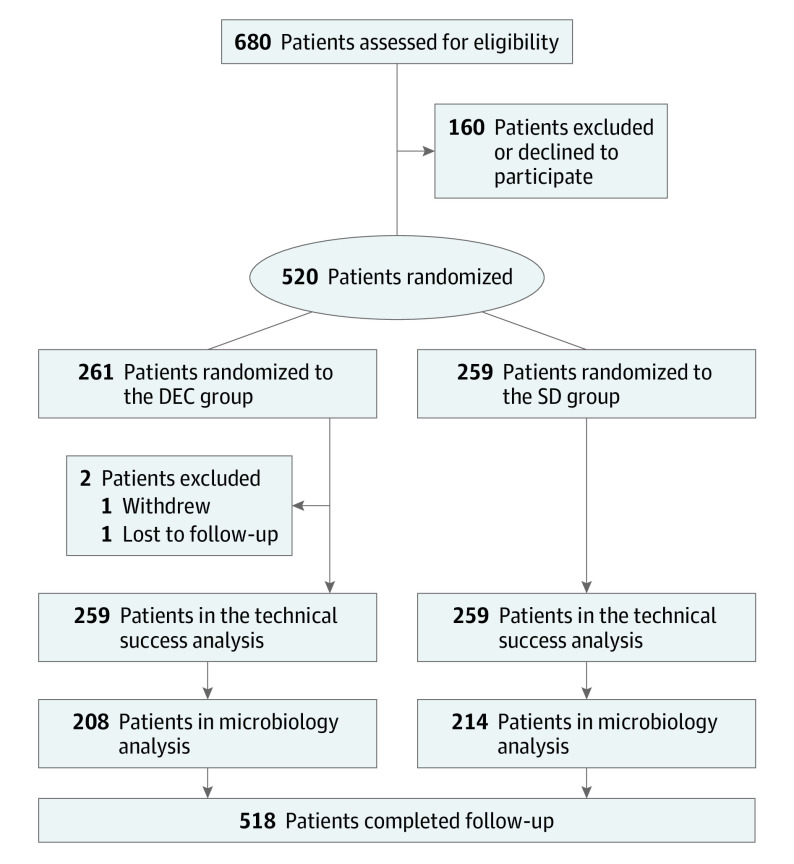
Flow Diagram of Screening, Randomization, Analyses of Coprimary Outcomes, and Follow-up DEC indicates disposable elevator cap; SD, standard duodenoscope.

From December 1, 2019, through February 28, 2022, including a pause in study recruitment due to the COVID-19 pandemic between March and September 2020, 520 patients undergoing ERCP were randomized. Two patients were randomized but not included in the final cohort; 1 withdrew consent, and the other was lost to follow-up as their periprocedural data were not captured. Of the 518 patients representing the final cohort, 259 were assigned to the disposable elevator cap group and 259 to the standard duodenoscope group. As planned, 208 patients in the disposable elevator cap group and 214 patients in the standard duodenoscope group had their duodenoscopes sampled after high-level disinfection. All 518 patients completed 30-day follow-up and were included in the primary analyses (Figure 1). No significant protocol violations occurred, and no missing data were observed. Neither attrition nor sample loss rates approached 10%.

The demographic, clinical, and procedural characteristics of the patients at baseline were similar between groups (Table 1). Patients had a mean (SD) age of 60.7 (17.0) years, and 49.8% were female. Their mean Charlson Comorbidity Index was 3.0. Procedures were of moderate complexity according to the ASGE grade (6.2% grade I, 70.1% grade II, 22.0% grade III, 1.7% grade IV).^29^

**Table 1.** Demographic, Clinical, and Procedural Characteristics of Study Participants

Characteristic	No. (%)		SMD
	Disposable elevator cap duodenoscope (n = 259)	Standard duodenoscope (n = 259)	
Age, mean (SD), y	60.3 (17.2)	61.1 (16.7)	−0.05
Sex			
Female	140 (54.1)	118 (45.6)	0.17
Male	119 (45.9)	141 (54.4)	
Charlson Comorbidity Index, mean (SD)	2.9 (2.4)	3.1 (2.8)	−0.09
Patient disposition			
Inpatient	114 (44.0)	101 (39.0)	0.10
Outpatient	145 (56.0)	158 (61.0)	
Trainee involvement			
Present	188 (72.6)	189 (73.0)	−0.01
Not present	71 (27.4)	70 (27.0)	
Previous ERCP			
No	135 (52.1)	127 (49.0)	−0.06
Yes	124 (47.9)	132 (51.0)	
Procedural indication			
Suspected or confirmed biliary stones	114 (44.0)	97 (37.5)	0.31
Suspected or confirmed malignant biliary obstruction	23 (8.9)	31 (12.0)	
Benign or anastomotic biliary stricture	19 (7.3)	9 (3.5)	
Cholangitis	10 (3.9)	5 (1.9)	
Repeat procedure including stent removal or exchange	51 (19.7)	58 (22.4)	
Other	42 (16.2)	59 (22.8)	
Procedural complexity using ASGE grade			
Grade I	16 (6.2)	16 (6.2)	0.03
Grade II	182 (70.3)	181 (69.9)	
Grade III	57 (22.0)	57 (22.0)	
Grade IV	4 (1.5)	5 (1.9)	
CBD cannulation achieved (in cases where CBD targeted)			
Yes	231 (95.9)	219 (93.6)	0.09
No	10 (4.1)	15 (6.4)	
Procedure time (intubation to extubation), median (IQR), min	14 (10-24)	17 (11-27)	NA

Abbreviations: ASGE, American Society for Gastrointestinal Endoscopy; CBD, common bile duct; ERCP, endoscopic retrograde cholangiopancreatography; NA, not applicable; SMD, standardized mean difference.

### Microbiology Outcomes

Persistent microbial contamination was detected in 11.2% of duodenoscopes in the standard duodenoscope arm and 3.8% of duodenoscopes in the disposable elevator cap duodenoscope arm (*P* = .004), corresponding to a RR of 0.34 (95% CI, 0.16-0.75) and NNT of 13.6 (95% CI, 8.1-42.7) to avoid 1 persistent microbial contamination event. Full results of microbiology outcomes, including frequency of duodenoscope use and repeated contamination, are provided in Table 2; the instrument channel was the predominant source of persistent microbial contamination in both groups. Of the 9 duodenoscopes in the standard duodenoscope group, all had at least 1 positive persistent microbial contamination result (100%), whereas 5 of 8 duodenoscopes in the disposable elevator cap group (62.5%) met this criterion. Detailed data on cultured microorganisms and growth patterns are provided in Table 3.

**Table 2.** Primary and Secondary Study Outcomes

Outcome	Disposable elevator cap duodenoscope			Standard duodenoscope			*P* value
	No. (%)	Scope ID	Occurrences/cases performed	No. (%)	Scope ID	Occurrences/cases performed	
**Microbiology outcomes**							
No.	208	NA	NA	214	NA	NA	NA
Persistent microbial contamination^a^							
Yes	8 (3.8)	NA	NA	24 (11.2)	NA	NA	.004
No	200 (96.2)	NA	NA	190 (88.8)	NA	NA	
No. of scopes with ≥10 CFU or growth of gram-negative bacteria							
In elevator region	2 (1.0)	NA	NA	2 (0.9)	NA	NA	.29
Within instrument channel	5 (2.4)	NA	NA	21 (9.8)	NA	NA	
In both areas	1 (0.5)	NA	NA	1 (0.5)	NA	NA	
Occurrence of ≥10 CFU or growth of gram-negative bacteria by scope (minimum of 10 cases performed)	NA	DEC-86	1/13	NA	SD-409	1/18	NA
		DEC-87	2/46		SD-410	1/17	
		DEC-89	0/30		SD-417	2/20	
		DEC-93	1/34		SD-724	2/21	
		DEC-117	0/10		SD-727	8/24	
		DEC-430	3/30		SD-729	1/23	
		DEC-842	0/14		SD-747	1/30	
		DEC-867	1/15		SD-1070	5/26	
**Technical and safety outcomes**							
No.	259	NA	NA	259	NA	NA	NA
Technical success							
Yes	245 (94.6)	NA	NA	235 (90.7)	NA	NA	.13
No	14 (5.4)	NA	NA	24 (9.3)	NA	NA	
Pancreatitis	8 (3.1)	NA	NA	9 (3.5)	NA	NA	>.99
Bleeding	5 (1.9)	NA	NA	3 (1.2)	NA	NA	.72
Perforation	1 (0.4)	NA	NA	0	NA	NA	>.99
Cholangitis	2 (0.8)	NA	NA	3 (1.2)	NA	NA	>.99
Cardiorespiratory events	4 (1.5)	NA	NA	2 (0.8)	NA	NA	.69
Death	1 (0.4)	NA	NA	1 (0.4)	NA	NA	>.99

Abbreviations: CFU, colony-forming unit; NA, not applicable.

^a^ Persistent microbial contamination defined as (1) growth of 10 or more colony forming units or (2) growth of any gram-negative organism, regardless of CFU. Some duodenoscopes grew multiple organisms.

**Table 3.** Species Identification of Organisms Isolated From Duodenoscopes Following High-level Disinfection and Associated Patterns of Growth^a^

Organism	Occurrence	CFU range	Typical habitat
**Disposable elevator cap duodenoscope (n = 208)**			
*Enterobacter *sp^b^	1	22	GI tract
*Enterococcus faecalis*	1	22	GI tract
*Obesumbacterium proteus*^b^ vs *Hafnia alvei*^b^^,^^c^	1	8	GI tract
*Staphylococcus epidermidis*	3	25-294	Skin
*Staphylococcus hominis*	3	25-256	Skin
*Staphylococcus capitis*	1	256	Skin
*Micrococcus luteus*	2	25-29	Skin
*Micrococcus cohnii*	1	29	Skin
*Micrococcus lylae*	1	29	Skin
*Micrococcus *sp	1	25	Skin
*Corynebacterium afermentans*	1	25	Skin
*Bacillus simplex*	1	22	Environment
*Pediococcus acidilactici*	1	22	Environment
*Stenotrophomonas maltophilia*	1	25	Environment
*Herbaspirillum huttiense* ^b^	1	6	Environment
*Paenibacillus peoriae*	1	17	Environment
Unknown^d^	3	17-22	NA
**Standard duodenoscope (n = 214)**			
*Enterococcus faecalis*	2	14-15	GI tract
*Enterococcus avium*	1	15	GI tract
*Escherichia coli* ^b^	1	150	GI tract
*Enterobacter* sp^b^	1	2	GI tract
*Klebsiella pneumoniae* ^b^	1	2368	GI tract
*Pantoea agglomerans* ^b^	1	34	GI tract
*Micrococcus luteus*	4	10-34	Skin
*Staphylococcus epidermidis*	4	11-43	Skin
*Staphylococcus hominis*	3	11-44	Skin
*Staphylococcus capitis*	3	10-292	Skin
*Staphylococcus lugdunensis*	2	11	Skin
*Staphylococcus caprae*	1	11	Skin
*Kocuria rhizophila*	1	15	Skin
*Bacillus paralicheniformis*	2	23-1632	Environment
*Bacillus altitudinis/pumilus*	2	31-36	Environment
*Paenibacillus provencensis*	1	10	Environment
*Paracoccus *sp AK26	1	34	Environment
*Priestia megaterium*	1	44	Environment
*Pseudomonas oryzihabitans* ^b^	1	2	Environment
*Gracilibacillus* sp	1	10	Environment
*Bacillus circulans*	1	11	Environment
*Bacillus flexus*	1	55	Environment
*Bacillus licheniformis*	1	189	Environment
*Bacillus megaterium*	1	34	Environment
*Bacillus simplex*	1	11	Environment
*Bacillus* sp	1	14	Environment
*Bacillus subtilis*	1	15	Environment
*Oceanobacillus kimchii*	1	15	Environment
Unknown^d^	4	44-189	NA

Abbreviations: CFU, colony-forming unit; GI, gastrointestinal; NA, not applicable.

^a^ Microorganisms were cultured from channel and elevator sample sites. All organisms listed if (a) growth determined at 10 CFUs or more at 72 hours and/or (b) gram-negative organism(s) isolated, regardless of CFUs. Multiple microorganisms may have been identified from the same duodenoscope.

^b^ Gram-negative bacteria.

^c^ Unable to differentiate.

^d^ Isolates could not be recovered after freezing, preventing identification.

### Technical and Safety Outcomes

Technical success with disposable elevator cap duodenoscopes was noninferior to that with standard duodenoscopes (94.6% vs 90.7%, *P* = .13). Patient tolerability was similar between groups, with no major differences in intraprocedural awareness or discomfort or postprocedural abdominal pain, throat pain, nausea, or distention.

There were no differences in infectious outcomes including cholangitis (0.8% vs 1.2%, *P* > .99) according to a recently updated classification system incorporating clinical criteria such as fever and leukocytosis.^22^ There were no differences in other adverse events, including pancreatitis (3.1% vs 3.5%, *P* > .99), bleeding (1.9% vs 1.2%, *P* = .72), perforation (0.4% vs 0.0%, *P* > .99), cardiorespiratory adverse events (1.5% vs 0.8%, *P* = .69), or 30-day mortality (0.4% vs 0.4%, *P* > .99). No adverse events were related to the study device. Full results of technical and safety outcomes are provided in Table 2.

### Subgroup Analyses

**Figure 2.**
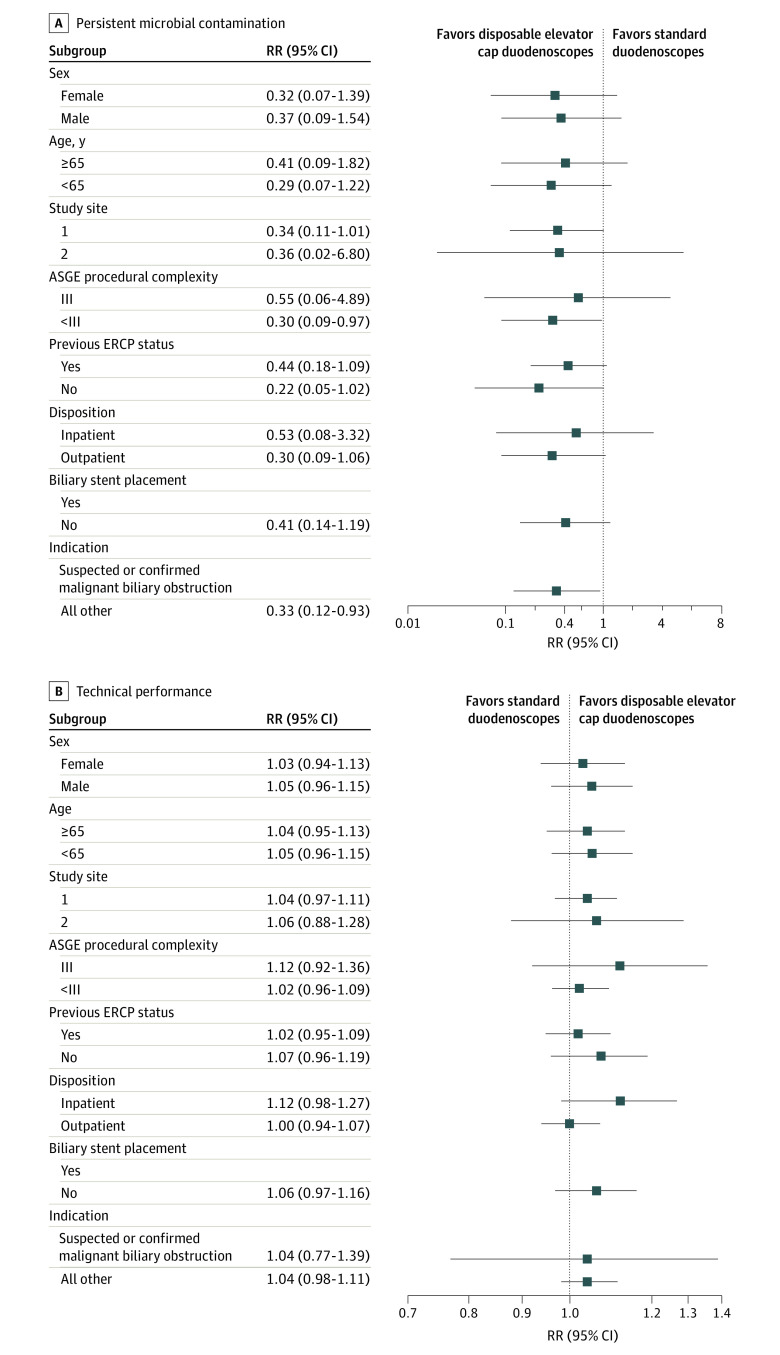
Subgroup Analyses for Coprimary Outcomes Relative risks are presented with standard duodenoscopes as the reference comparator. ASGE indicates American Society for Gastrointestinal Endoscopy; ERCP, endoscopic retrograde cholangiopancreatography; RR, relative risk. Note: among the ERCPs with biliary stent placement, technical success was 100% in both arms, and there were 0 cases of persistent contamination in the disposable elevator cap arm—therefore, no point estimates are provided for this subgroup given that RRs and/or CIs are inestimable.

The results of all performed post hoc subgroup analyses are provided in Figure 2. Without exception, point estimates for all subgroups favored reduced persistent microbial contamination for all subgroups with disposable elevator cap duodenoscopes compared with standard duodenoscopes. Similarly, point estimates for all subgroups pointed to similar or greater technical success for disposable elevator cap duodenoscopes compared with standard duodenoscopes. The results of the sensitivity analysis correcting for multiple comparisons and reporting the 95% CIs are presented in plots in eFigure 3 in Supplement 1.

## Discussion

In this randomized clinical trial, disposable elevator cap duodenoscopes exhibited lower rates of persistent microbial contamination after high-level disinfection compared with standard duodenoscopes while maintaining noninferior rates of technical performance for ERCP. There were no differences between study groups for adverse events, ease of duodenoscope use, or patient tolerability. These results were consistent across all clinically relevant subgroups.

When considering that duodenoscope-related infections are estimated to occur at rates of approximately 0.01%,^27^ a sample size of over 400 000 patients would be required for an adequately powered randomized trial evaluating this clinical outcome. Therefore, persistent microbial contamination was chosen as a surrogate outcome measure given (1) it is a required step in the sequence leading to duodenoscope-related transmission (index patient colonized, colonized patient gets ERCP, duodenoscope is colonized, next patient has ERCP with colonized scope, colonized patient becomes clinically infected),^26^ (2) it is measurable using standardized microbiologic protocols,^23^ and (3) it is the most predictive end point available when compared with other techniques.^30^

A 2022 study by Ridtitid et al^31^ demonstrated a decreased burden of organic residue with disposable elevator cap duodenoscopes compared with standard duodenoscopes when assessed by point-of-care ATP scanning. However, ATP testing correlates poorly with cultures,^32^ with another study demonstrating that greater than 50% of duodenoscopes had persistent microbial contamination following negative ATP testing.^30^ These findings should cast some doubt over the significance and generalizability of the study by Ridtitid et al^31^ when interpreted in isolation. All duodenoscopes in our study were required to pass ATP testing prior to microbiologic analysis; therefore, our results further demonstrate poor correlation between ATP testing and persistent microbial contamination. A possible reason is that ATP testing may miss contamination of the instrument channel, which represented the majority of persistent microbial contamination in our study. Duodenoscopes that have passed ATP testing after high-level disinfection are still at risk of contamination.

The findings of our study support the FDA recommendation to transition to duodenoscopes with novel designs.^14^ Although entirely disposable duodenoscopes offer the ability to completely eliminate persistent microbial contamination, the high costs, lower technical performance, and environmental effects all limit the widespread use of these devices.^15,16^ Duodenoscopes with a disposable elevator cap offer a potential solution for substantially reducing persistent microbial contamination that is scalable, with an incremental cost of approximately $50, or 3% to 7% of overall procedural costs.^33^ A 2022 cost utility analysis also supports the use of disposable elevator cap duodenoscopes.^34^ The prevention of duodenoscope-related infections, however, also requires consideration of additional factors, including personnel training and communication between those involved in the procedures.^35^

The fact that the majority of episodes of persistent microbial contamination originated from the instrument channel in both study groups underscores the complex multiple mechanisms involved with microbial contamination of duodenoscopes.^36^ One possibility is that the disposable elevator cap design permits more effective decontamination of the entire duodenoscope, enabling more efficient sterilization of the channel. Another possibility is that the nondisposable part of the elevator region constitutes a potential reservoir for microbes, which can contaminate the channel or result in biofilms, polymicrobial structures that can adhere to surfaces, withstand shear forces, and resist chemical disinfection.

Although the formation of bacterial biofilms may explain why some duodenoscopes sporadically have repeated persistent microbial contamination and not others, cultured microorganisms differed within the same duodenoscope, arguing against this hypothesis. Instead, undetectable areas of microtrauma within the instrument channel could be responsible for enabling recurrent persistent microbial contamination,^36^ with the difference in the elevator caps between the 2 types of duodenoscope mechanisms being a potentially important differentiating factor for the introduction of pathogens. To minimize potential bias related to individual instruments, all study duodenoscopes were between 12 and 24 months old and had all passed routine maintenance. Although the age of the duodenoscopes represents a potential source of bias, this is unlikely to have resulted in differences in contamination rates given that a prior study demonstrated instrument channel scratches and/or shredding of the scope lining within 4 weeks in new endoscopes.^37^ If they were functioning properly, our study duodenoscopes did not undergo routine evaluation for microdefects, consistent with usual clinical practice.

In our study, there were no device-related adverse events. However, a recent analysis of the FDA Manufacturer and User Facility Device Experience database reported endcap detachments and fractures,^38^ that although rare, highlight the importance of (1) having personnel receive instruction to ensure optimal use and (2) using disposable elevator caps within their designated shelf lives.

### Limitations

Our trial has limitations. One limitation is the difficulty associated with generalizing local microbial growth patterns outside of the 2 study centers in Canada. However, the rates of persistent microbial contamination and the microbiological taxonomy were similar to those in a 2018 Dutch study of digestive tract bacteria in duodenoscopes,^12^ with the caveat that we observed a lower rate of contamination with enteric gram-negative bacteria. A second limitation is that most of the ERCPs in our trial were of moderate procedural complexity, with only 23.7% of procedures being an ASGE grade III or IV. However, the point estimates of technical performance were similar in this subgroup to the point estimates for less technically demanding procedures. Third, we did not collect data on patient colonization or local antimicrobial resistance patterns. Finally, even though the noninferiority margin of 7% for technical success could be considered too high given the low risk of duodenoscope-related infections, technical success was assessed blindly and the point estimate for technical success was higher for disposable elevator cap duodenoscopes (95% vs 91%). This finding suggests that a meaningful difference between the 2 types of endoscopes with regard to technical performance is unlikely.

## Conclusions

In this randomized clinical trial, we demonstrated that disposable elevator cap duodenoscopes exhibited lower persistent microbial contamination rates following high-level disinfection compared with standard duodenoscopes while maintaining a noninferior technical performance of ERCP and similar safety outcomes.

## Notes

Supplement 1. **Figure 1.** Diagram illustrating duodenoscope designs and sample acquisition areas **eTable 1.** CONSORT 2010 Checklist of information to include when reporting a randomised trial **eTable 2.** Summary of protocol versions and changes **eMethods 1.** Detailed study eligibility criteria **eTable 3.** Definitions of technical success by ERCP indication **eMethods 2.** Duodenoscope microbiological sample acquisition protocol **eFigure 2.** Details of study enrolment across both sites **eMethods 3.** Filtration, plating, and identification protocol **eFigure 3.** Post hoc subgroup analyses corrected for multiple comparisons, with 95% confidence intervals

Supplement 2. Trial Protocol

Supplement 3. Data Sharing Statement
