# Supplementary material for: Effect of Disposable Elevator Cap Duodenoscopes on Persistent Microbial Contamination and Technical Performance of Endoscopic Retrograde Cholangiopancreatography: The ICECAP Randomized Clinical Trial
Source: JAMA Intern Med. 2023 Jan 23;183(3):191–200. doi: 10.1001/jamainternmed.2022.6394 (PMC9871945; doi:10.1001/jamainternmed.2022.6394)
Supplement: Supplement 1. — Figure 1. Diagram illustrating duodenoscope designs and sample acquisition areas eTable 1. CONSORT 2010 Checklist of information to include when reporting a randomised trial eTable 2. Summary of protocol versions and changes eMethods 1. Detailed study eligibility criteria eTable 3. Definitions of technical success by ERCP indication eMethods 2. Duodenoscope microbiological sample acquisition protocol eFigure 2. Details of study enrolment across both sites eMethods 3. Filtration, plating, and identification protocol eFigure 3. Post hoc subgroup analyses corrected for multiple comparisons, with 95% confidence intervals [file jamainternmed-e226394-s001.pdf]

Supplemental Online Content

Forbes N, Elmunzer BJ, Allain T, et al. Effect of disposable elevator cap duodenoscopes on persistent microbial contamination and technical performance of endoscopic retrograde cholangiopancreatography: the ICECAP randomized clinical trial. *JAMA Intern Med.* Published online January 23, 2023. doi:10.1001/jamainternmed.2022.6394

eFigure 1. Diagram illustrating duodenoscope designs and sample acquisition areas

eTable 1. CONSORT 2010 Checklist of information to include when reporting a randomised trial

eTable 2. Summary of protocol versions and changes

eMethods 1. Detailed study eligibility criteria

eTable 3. Definitions of technical success by ERCP indication

eMethods 2. Duodenoscope microbiological sample acquisition protocol

eFigure 2. Details of study enrolment across both sites

eMethods 3. Filtration, plating, and identification protocol

eFigure 3. Post hoc subgroup analyses corrected for multiple comparisons, with 95% confidence intervals

This supplemental material has been provided by the authors to give readers additional information about their work.

© 2023 Forbes N et al. *JAMA Internal Medicine.*

eFigure 1. Diagram illustrating duodenoscope designs and sample acquisition areas.

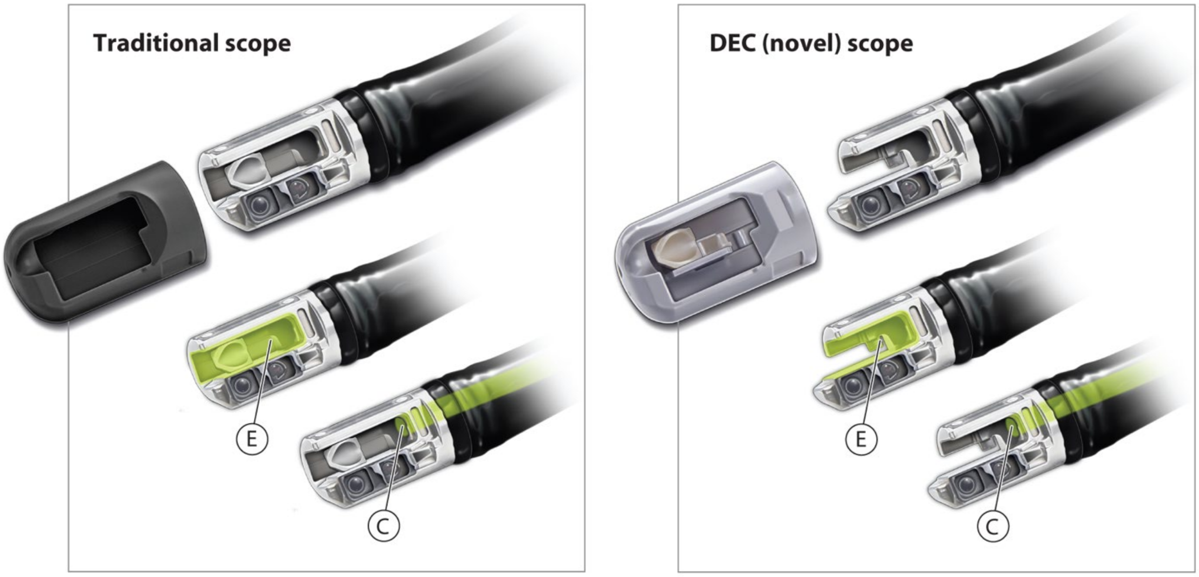

E, elevator swabbing area; C, channel swabbing area.

© 2023 Forbes N et al. *JAMA Internal Medicine.*

eTable 1. CONSORT 2010 Checklist of information to include when reporting a randomized trial.

Section/Topic	Item No	Checklist item	Reported on page No
**Title and abstract**			
	1a	Identification as a randomised trial in the title	1
	1b	Structured summary of trial design, methods, results, and conclusions (for specific guidance see CONSORT for abstracts)	4-5
**Introduction**			
Background and objectives	2a	Scientific background and explanation of rationale	6
	2b	Specific objectives or hypotheses	6
**Methods**			
Trial design	3a	Description of trial design (such as parallel, factorial) including allocation ratio	6-7
	3b	Important changes to methods after trial commencement (such as eligibility criteria), with reasons	N/A
Participants	4a	Eligibility criteria for participants	7, S5
	4b	Settings and locations where the data were collected	6-7
Interventions	5	The interventions for each group with sufficient details to allow replication, including how and when they were actually administered	7
Outcomes	6a	Completely defined pre-specified primary and secondary outcome measures, including how and when they were assessed	8-9
	6b	Any changes to trial outcomes after the trial commenced, with reasons	N/A
Sample size	7a	How sample size was determined	9-10
	7b	When applicable, explanation of any interim analyses and stopping guidelines	N/A
Randomisation:			
Sequence generation	8a	Method used to generate the random allocation sequence	7
	8b	Type of randomisation; details of any restriction (such as blocking and block size)	6-7
Allocation concealment mechanism	9	Mechanism used to implement the random allocation sequence (such as sequentially numbered containers), describing any steps taken to conceal the sequence until interventions were assigned	7
Implementation	10	Who generated the random allocation sequence, who enrolled participants, and who assigned participants to interventions	7

© 2023 Forbes N et al. *JAMA Internal Medicine.*

Blinding	11a	If done, who was blinded after assignment to interventions (for example, participants, care providers, those assessing outcomes) and how	7
	11b	If relevant, description of the similarity of interventions	7
Statistical methods	12a	Statistical methods used to compare groups for primary and secondary outcomes	10
	12b	Methods for additional analyses, such as subgroup analyses and adjusted analyses	10
**Results**			
Participant flow (a diagram is strongly recommended)	13a	For each group, the numbers of participants who were randomly assigned, received intended treatment, and were analysed for the primary outcome	11
	13b	For each group, losses and exclusions after randomisation, together with reasons	11
Recruitment	14a	Dates defining the periods of recruitment and follow-up	11
	14b	Why the trial ended or was stopped	N/A
Baseline data	15	A table showing baseline demographic and clinical characteristics for each group	16
Numbers analysed	16	For each group, number of participants (denominator) included in each analysis and whether the analysis was by original assigned groups	Fig1
Outcomes and estimation	17a	For each primary and secondary outcome, results for each group, and the estimated effect size and its precision (such as 95% confidence interval)	11-12
	17b	For binary outcomes, presentation of both absolute and relative effect sizes is recommended	11-12
Ancillary analyses	18	Results of any other analyses performed, including subgroup analyses and adjusted analyses, distinguishing pre-specified from exploratory	11-12
Harms	19	All important harms or unintended effects in each group (for specific guidance see CONSORT for harms)	11-12
**Discussion**			
Limitations	20	Trial limitations, addressing sources of potential bias, imprecision, and, if relevant, multiplicity of analyses	13-15
Generalisability	21	Generalisability (external validity, applicability) of the trial findings	13-15
Interpretation	22	Interpretation consistent with results, balancing benefits and harms, and considering other relevant evidence	13-15
**Other information**			
Registration	23	Registration number and name of trial registry	5
Protocol	24	Where the full trial protocol can be accessed, if available	7
Funding	25	Sources of funding and other support (such as supply of drugs), role of funders	21-22

© 2023 Forbes N et al. *JAMA Internal Medicine.*

eTable 2. Summary of protocol versions and changes.

**Protocol Version/ Name**	**Protocol Date**	**Major Changes from Prior Version**
VERSION A ('v3.0')	2019-06-19	• N/A (original working draft)
VERSION B ('v6.1')	2019-07-16	• Underwent revision and edits by all co-investigators<br>• Eligibility criteria finalized<br>• Primary outcomes finalized<br>• Initial protocol submitted to University of Calgary REB
VERSION C ('v6.2')	2019-07-31	• First protocol approved by University of Calgary REB (no major changes from prior version)
VERSION D ('v7.0')	2019-12-01	• Addition of MALDI analyses for any isolated samples with significant or uncertain growth patterns<br>• Given hospital reprocessing change to double HLD cycles for all endoscopes, threshold for CFU to qualify as positive outcome changed to 10 (from 20) by study steering committee (no patients recruited yet)
VERSION E ('v8.0')	2020-11-23	• Addition of second study site (Kingston, ON, Canada)

© 2023 Forbes N et al. *JAMA Internal Medicine.*

eMethods 1. Detailed study eligibility criteria.

Inclusion Criteria

Patients will be required to meet *all* of the following inclusion criteria in order to be eligible for study participation:

- age ≥ 18 years;
- ability to give informed consent to involvement;
- requirement for ERCP to be performed for any indication.

Exclusion Criteria

Patients meeting *any* of the following exclusion criteria will not be eligible for study participation:

- age < 18 years;
- inability or unwillingness to provide informed consent;
- standard contraindications to ERCP;
- pregnant status or breastfeeding mother;
- inability to successfully complete an ERCP procedure under conscious sedation;
- out-of-province status;
- incarceration.

© 2023 Forbes N et al. *JAMA Internal Medicine.*

eTable 3. Definitions of technical success by ERCP indication.[19]

Indication for ERCP	Definition(s) of Technical Success
Suspected or confirmed bile duct stone(s)	Extraction of stone(s)<br>OR<br>CBD clearance based on absence of filling defects on occlusion cholangiogram<br>*If difficult biliary stones are encountered during procedure, use 'Difficult biliary stone(s)' indication below, and above definition does not apply
Difficult biliary stone(s) – any of:<br>• One or more stone(s) ≥ 15 mm<br>• Barrel or other unusual shape<br>• Multiple (4 or more stones)<br>• Impacted stone(s)<br>• Intrahepatic or cystic duct stone(s)<br>• Stricture below stone(s)	Extraction of stone(s)<br>OR<br>CBD clearance based on absence of filling defects on occlusion cholangiogram<br>OR<br>Stenting of CBD as part of future plan to clear duct
Biliary stricture (benign or malignant)	Successful placement of stent with proximal margin proximal to stricture<br>OR<br>Successful dilatation of stricture
Cholangioscopy or pancreatoscopy	Successful cholangioscopic or pancreatoscopic visualization of area of interest
Chronic pancreatitis, pancreatic stone(s) and/or pancreatic stricture(s)	Successful cannulation of main pancreatic duct (PD)<br>AND AT LEAST 1 OF:<br>Pancreatic sphincterotomy<br>Stenting or dilatation of PD<br>Extraction of PD stone(s)
Pancreas divisum	Successful minor papilla cannulation<br>AND<br>Successful pancreatic sphincterotomy
Stent removal or exchange	Successful removal and/or exchange of stent(s)
Treatment of peri-ampullary bleeding	Successful endoscopic hemostasis
Sphincter of Oddi dysfunction	Successful biliary sphincterotomy

© 2023 Forbes N et al. *JAMA Internal Medicine.*

eMethods 2. Duodenoscope microbiological sample acquisition protocol.

1. Materials (Checklist)

- Hand sanitizer
- Lab gown
- Fluid-resistant sterile gown
- Face mask
- Two transfer pipets
- Sterile gloves
- Bouffant caps for hair
- Surface disinfectant for countertop (Virkon and EtOH 70%)
- Sterile fluid-resistant pad or drape
- Sterile collection container (empty; labelled either Elevator or Channel)
- Sterile Dey-Engley (DE) Broth (labelled either Elevator or Channel)
    - Elevator: 30 mL; channel: 45 mL
- Sampling fluid: Sterile deionized water (labelled either Elevator or Channel)
    - Elevator: 30 mL; channel: 45 mL
- Sterile cleaning brushes Pentax Cleaning Brush CS - C13A (Double head single-use cleaning brush for elevator) and CS5522A (Yellow single-use cleaning brush for channel)
- Pliers and scissors
- Sterile 50 mL syringes (2 syringes with 0.8 um filters)
- A countertop or table long enough to lay the scope out for inspection
- Ice pack/foam box
- Permanent marker

2. Sample Preparation (step by step):

1. Label sterile container appropriately with patient number (empty bottles and media bottles).
2. Apply mask and Bouffant cap.
3. Perform hand hygiene.
4. Apply gown.
5. Perform hand hygiene.
6. Put gloves on.
7. Disinfect work area with Virkon and EtOH (wait 2 minutes before swiping).
8. Place sterile drape on work surface and place duodenoscope on sterile pad without contact with distal tip (handle can be placed outside of the drape).
   NB: for the pilot, please tape 2 sterile drapes together.
9. Clean and wipe all packages and tools with EtOH.
10. Perform visual inspection; if soil/biofilm observed notify on a list (attached): Yes or No

© 2023 Forbes N et al. *JAMA Internal Medicine.*

3. Sampling

3.1. Elevator Recess Sampling (label: Elevator)

1. Open sterile pipette package; open sterile water container.
2. Fill sterile pipette with 1-2 mL sterile water (approximately).
3. Place cap back on sterile water container.
4. Lower elevator lever (flat position).
5. Hold distal end parallel to draped surface.
6. Place open sterile container underneath distal end.
7. Apply 1-2 mL sterile water to elevator recess with pipette and allow fluid to drain into collection container (**first flush**) – SEE APPENDIX PICTURES.
8. Raise elevator (up position).
9. Repeat steps 1-3 and 5-7 above (**second flush**). <u>Place the transfer pipet back in the package for further use (step 17).</u>
10. Open sterile CS - C13A (Double head cleaning brush; **green brush**).
    Clean package with EtOH, open longitudinally, and pick up with forceps to avoid touching with gloves.
11. Remove brush from package and hand it to second technician.
12. Raise elevator to upper position.

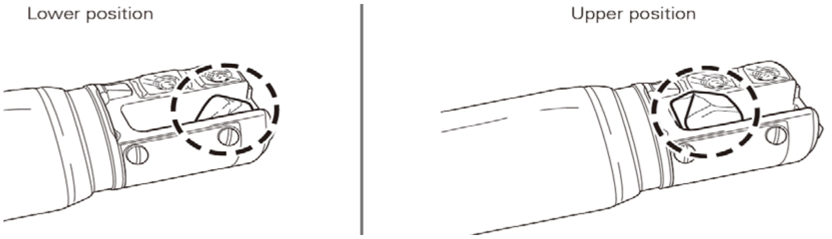

13. Dip brush in sterile water, moisten small end of brush, and brush along the walls of the elevator chamber crock-wise and behind the elevator (thoroughly).

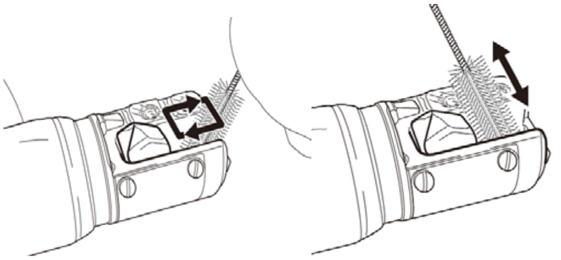

© 2023 Forbes N et al. *JAMA Internal Medicine.*

14. Lower elevator to lower position & brush in (a,b,c) pattern (thoroughly).

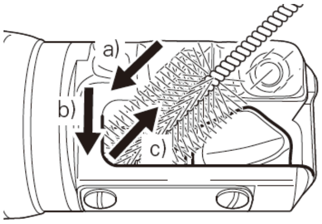

15. Cut the brush with clean pliers (sterilized with EtOH).
16. Place brush into sample container (collection container) and close the lid. (DON'T USE THE BIG BRUSH).
17. Repeat steps a to g two times (with the same transfer pipet).

a) Open sterile pipette package; open sterile water container.
b) Fill sterile pipette with 1-2 mL sterile water (approximately).
c) Place cap back on sterile water container.
d) Lower elevator lever (flat position).
e) Hold distal end parallel to draped surface.
f) Place open sterile container underneath distal end.
g) Apply 1-2 mL sterile water to elevator recess with pipette and allow fluid to drain into collection container (**flush**).

18. **Addition of neutralizer solution and transport preparation**

a) Pour the remaining water in the collection container and add 30 mL of DE broth (**labelled elevator**) to sample (entire content).
b) Tightly close lid of sample container and properly place sample into transport container.

Place parafilm on for transportation.
Ensure sample will be maintained at proper temperature range.

3.2. Instrument Channel Sampling (Label Channel)

1. Open (2) 50 mL syringes, place 0.8 μm filter on to avoid spillage, then remove the plunger and pour in 20 mL sterile water and place plunger back on syringes. Syringe can be placed on the sterile drape (syringe won't fit in the container).
2. Elevate control handle to get scope as vertical as possible.
3. Person A hands syringe to Person B and holds distal tip over sample collection container.

Important: screw the syringe onto the channel (syringe can't move).

4. Flush instrument channel via biopsy port with 20 mL, which is collected into sample container by gravity.

5. Fill syringe with air and push any residual fluid out and into collection container.
6. Place scope on sterile drape.
7. Remove brush CS5522A (yellow brush) from package.
8. First technician - hold scope vertically while second technician inserts brush into biopsy port about 3 inches then transfers the brush handle to first technician - SEE APPENDIX PICTURES.
9. Hold container below distal tip to capture any fluid that exits with the brush; SEE APPENDIX PICTURES.
10. Push brush through the scope; - SEE APPENDIX PICTURES.
11. First technician - use scissors/pliers to cut head of brush off into the sample container; second technician closes the lid.
12. Pull brush back up and out the biopsy port (without contaminating).
13. Repeat steps 1-5 with second syringe filled with 25 mL sterile water (rest of the bottle).
14. Addition of neutralizer solution and transport preparation

a) Pour the remaining water in the collection container and add 45 mL of DE broth (labelled elevator) to sample (entire content).

b) Tightly close lid of sample container and properly place sample into transport container.

Place parafilm on for transportation.

Ensure sample will be maintained at proper temperature range

© 2023 Forbes N et al. *JAMA Internal Medicine.*

eFigure 2. Details of study enrolment across both sites.

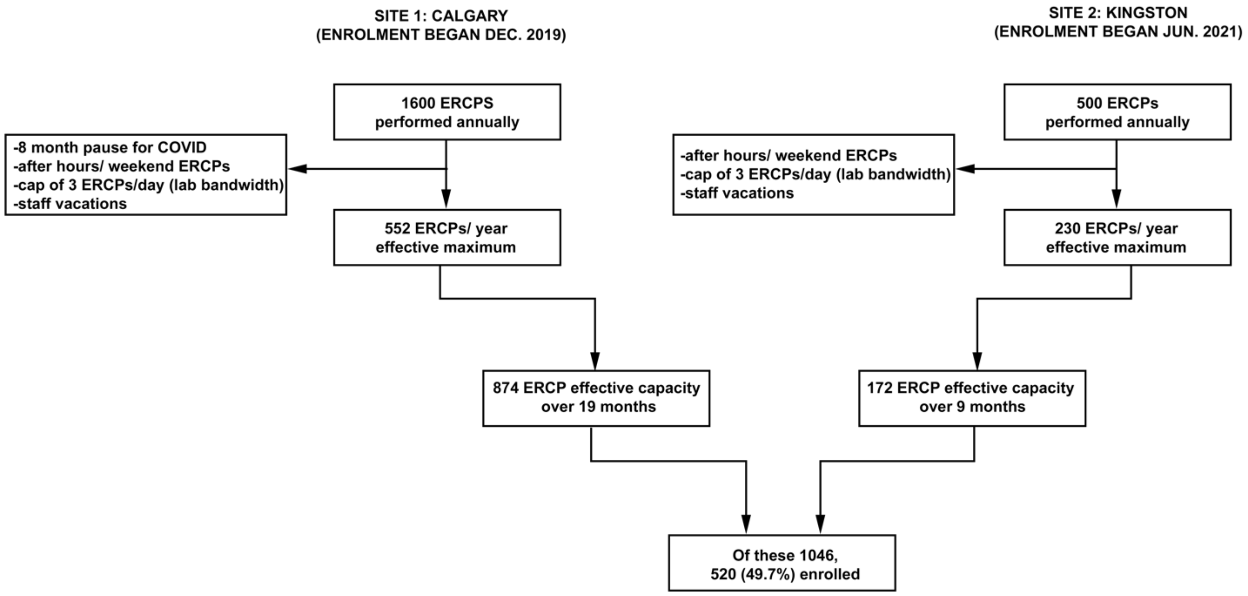

© 2023 Forbes N et al. *JAMA Internal Medicine.*

eMethods 3. Filtration, plating, and identification protocol.

Filtration

1. Ensure the 6-funnel filtration unit is clean (without gross contamination) and functioning properly.
2. Magnetic funnels previously sanitized and cleaned are capped with aluminum foil, completely wrapped in autoclave cloth and autoclaved BEFORE filtration.
3. Allow time of funnels to cool prior to use.
4. Carefully unwrap funnels and place inside 6-funnel filtration unit with rubber adaptor.
5. For stainless steel funnels flame BEFORE and AFTER filtration as described below.
6. Using 6-funnel filtration unit with valve turned off, spritz with EtOH, and carefully light. Wait until it burns out and cap. Allow time to ensure funnel and platform are dry and cool.
7. Vortex samples for 30 sec before filtration step.
8. Carefully (and sterile) with EtOH-flamed forceps, place 0.45 μM filter grid-side-up. Place funnel back carefully, wrap to seal with parafilm and secure with clamps for stainless steel funnels.
9. Turn on vacuum pump and valve for specific funnel, then carefully pour sample into funnel until no visible liquid remains. Turn valve off and take filter out with sterile forceps, placing grid-side-up slowly one side at a time on CBA, avoiding bubbles underneath.
10. Incubate for 72 h 37 °C in humid incubator and check for colonies.
11. Place the plates for an additional 48 h (5 d total) as per FDA requirement and look for additional bacterial colonies.

Picking Colonies and Identification

1. Report total colony count in CFU/mL on blood agar.
2. Pick colonies onto CBA, CNA, and MAC scratch plate to test for growth; Gram positives should grow on CNA and Gram negatives should grow on MAC. Each isolate should be serially plated 3× on CBA to make sure culture is pure.

© 2023 Forbes N et al. *JAMA Internal Medicine.*

3. Identify Gram negative bacilli (GNB) by growth on MAC and/or Gram stain. Non-GNB isolates can be reported as CFU/mL and can be grouped for identification. Yeast will be separately identified and detailed.
4. Formal identification of GNB will be performed at a later date, using MALDI or 16S rRNA gene PCR.
   a. Freeze down 2 tubes for each purified isolate in BHI 25% glycerol cryo tubes and store at −80°C for future use, or to send for MALDI and biobank. Alternatively, stabs can be used to send isolates for MALDI using 1 mL LB 0.7% agar in cryo tubes.
   b. For 16S PCR, use a purified fresh colony.

MALDI identification

1. MALDI identification was performed at the Kingston Health Sciences Centre Clinical Microbiology Laboratory.

16S PCR and Sanger sequencing

1. Use 5 μL of the stored isolates in BHI 25% glycerol cyro tubes for 16S PCR.
2. 5 μL 10× PCR buffer, 1.5 μL $MgCl_2$ (50 mM), 1 μL dNTPs (10 mM), 1 μL each F and R primers (10 pmol), 38.25 μL ddH_2O, 0.25 μL Taq Polymerase, and 5 μL DNA.
3. Place in PCR machine and heat 98 °C 10 min, cool to 4 °C and centrifuge 10 min.
4. PCR cycle conditions: Initial denaturation 2 min at 94 °C, 32 cycles of denaturation 1 min at 94 °C annealing 1 min at 56 °C, extension 2 min at 72 °C, final extension 5 min at 72 °C, hold at 12°C.

Primers: 16S Amplicon PCR Forward Primer = 5' TCGTCGGCAGCGTCAGATGTGTATAAGAGACAGCCTACGGGNGGCWGCAG 16S Amplicon PCR Reverse Primer = 5' GTCTCGTGGGCTCGGAGATGTGTATAAGAGACAGGACTACHVGGGTATCTAATCC

5. Confirm PCR amplicons using 1% agarose gel electrophoresis.
6. Purify PCR amplicons using Beckman Coulter© AMPure XP beads, 80% ethanol and Tris wash.

© 2023 Forbes N et al. *JAMA Internal Medicine.*

7. Measure DNA concentration using Qubit 2.0 Fluorometer.
8. Send for Sanger sequencing: 60-100ng PCR, 3 pmol primer, and ddH_2O in final volume 7.7 µL. Include 0.5 µL of either the F or R primer in each sequencing reaction, one for the forward and another for reverse.

© 2023 Forbes N et al. *JAMA Internal Medicine.*

eFigure 3. *Post hoc* subgroup analyses corrected for multiple comparisons, with 95% confidence intervals.

a)

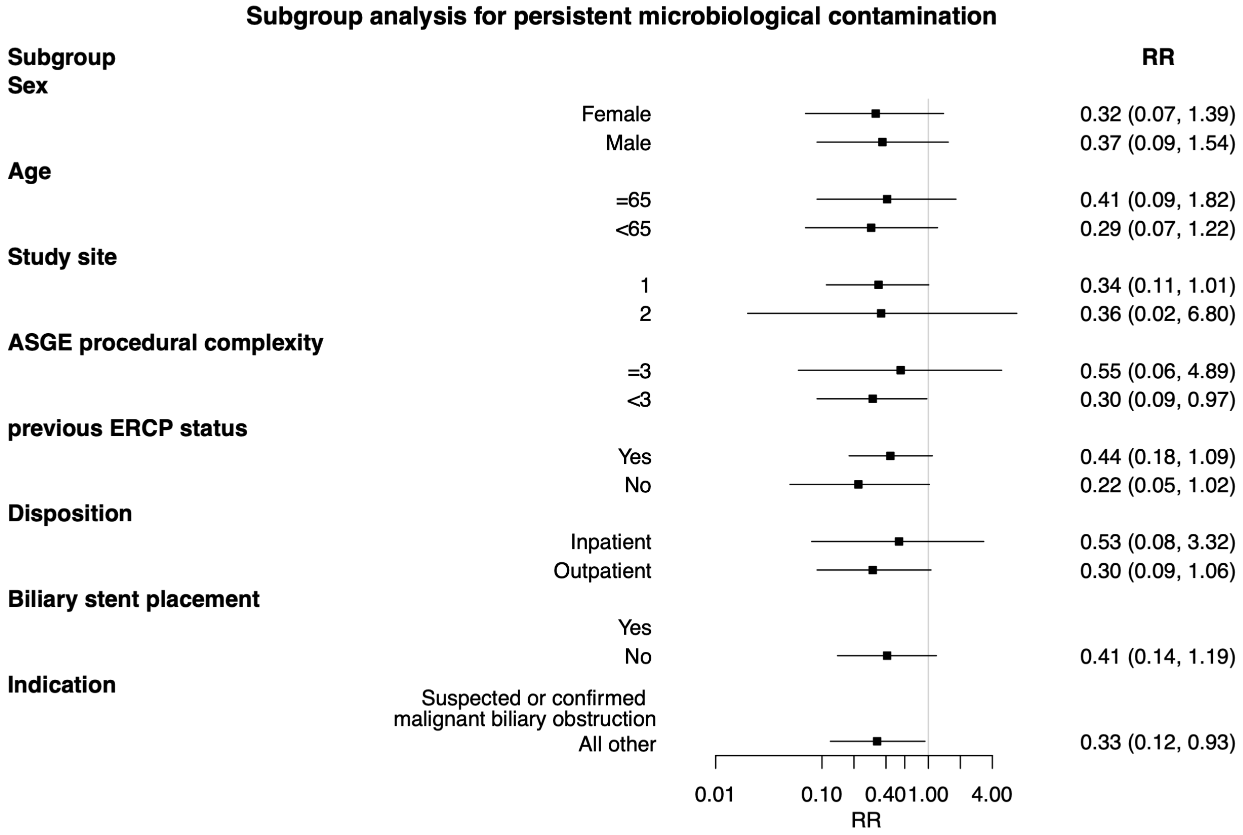

© 2023 Forbes N et al. *JAMA Internal Medicine.*

b)

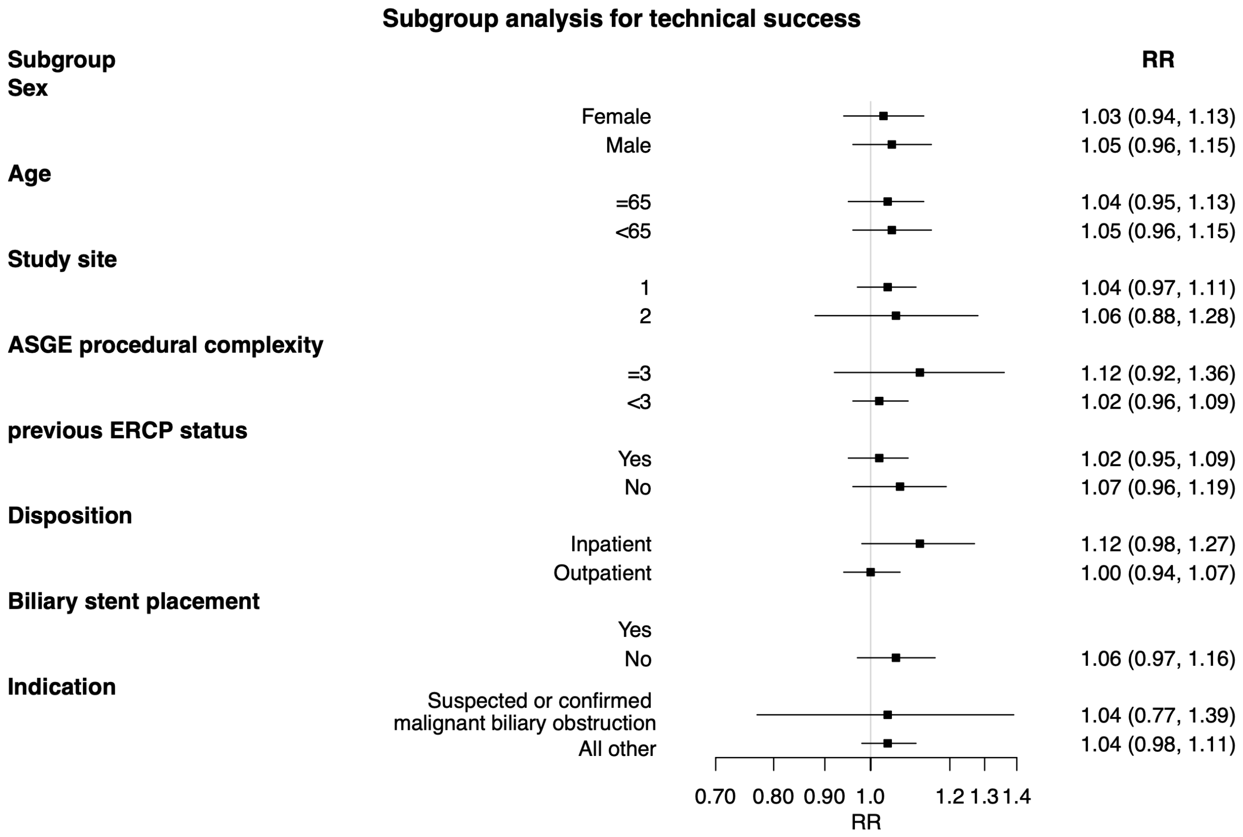

© 2023 Forbes N et al. *JAMA Internal Medicine.*
