# Supplementary material for: Effect of Disposable Elevator Cap Duodenoscopes on Persistent Microbial Contamination and Technical Performance of Endoscopic Retrograde Cholangiopancreatography: The ICECAP Randomized Clinical Trial
Source: JAMA Intern Med. 2023 Jan 23;183(3):191–200. doi: 10.1001/jamainternmed.2022.6394 (PMC9871945; doi:10.1001/jamainternmed.2022.6394)
Supplement: Supplement 2. — Trial Protocol [file jamainternmed-e226394-s002.pdf]

# STUDY PROTOCOL

**Clinical Efficacy and Infection Risk of ERCP using a Novel Duodenoscope with a Disposable Cap: a Randomized Controlled Trial**

Short Title: Infection Control in ERCP using a Duodenoscope with a Disposable Cap (ICECAP)

Principal Investigator: Nauzer Forbes MD MSc[1]
Clinical Assistant Professor
Division of Gastroenterology
Cumming School of Medicine, University of Calgary
TRW 6D19, 3280 Hospital Dr. NW
T2N 4Z6 Calgary, AB
p 403.592.5089 f 403.592.5090
nauzer.forbes@ucalgary.ca

Co-investigators: B Joseph Elmunzer MD MSc[2]
Steven J Heitman MD MSc[1]
Andre G Buret PhD[1]
Thibault Allain PhD[1]
Sydney Bass MD[1]
Paul Belletrutti MD[1]
Martin J Cole MD[1]
Emmanuel Gonzalez-Moreno MD[1]
Ahmed Kayal MD[1]
Puja Kumar MD[1]
Rachid Mohamed MD[1]
Christian Turbide MD[1]

Affiliations: [1]University of Calgary, Calgary, Alberta, Canada
[2]Medical University of South Carolina, Charleston, South Carolina, USA

Protocol Version: 6.2

## 1. Introduction

### *1.1. Background*

Endoscopic retrograde cholangiopancreatography (ERCP) is well recognized as an important therapeutic modality for biliary and/or pancreatic pathology. There are various established roles of ERCP, including: the first-line modality for management of choledocholithiasis,[1] the first-line modality for decompressive management of pancreatico-biliary strictures,[2] the first-line modality (in conjunction with cholangioscopy) for evaluation and/or treatment of proximal biliary neoplasia,[3] and an important modality in the treatment of several benign pancreatico-biliary disorders, including Sphincter of Oddi dysfunction and pancreas divisum.[4] In conjunction with other endoscopic, surgical and radiographic techniques, ERCP is therefore a cornerstone in the management of pancreatico-biliary disease. While effective, ERCP has several well-established associated adverse events. These include post-ERCP pancreatitis (PEP), bleeding, cholangitis, cholecystitis, perforation, and cardiopulmonary events.[5]

ERCP is universally performed using a highly specialized type of flexible endoscope called a duodenoscope. Rather than having a forward-facing camera at the end of the scope (which is typical of gastroscopes and colonoscopes), duodenoscopes have a side-viewing camera close to the distal tip that faces perpendicularly to the long axis of the scope. A representation of a typical duodenoscope tip is shown in Figure 1. The unique design of a duodenoscope affords direct visualization of the major and/or minor papillae, which is a requisite for successful biliary and/or pancreatic cannulation and instrumentation. In addition, a recess in the tip of the duodenoscope houses an elevator, a device designed to assist with fine tip control of pancreatico-biliary cannulation and extraction devices.

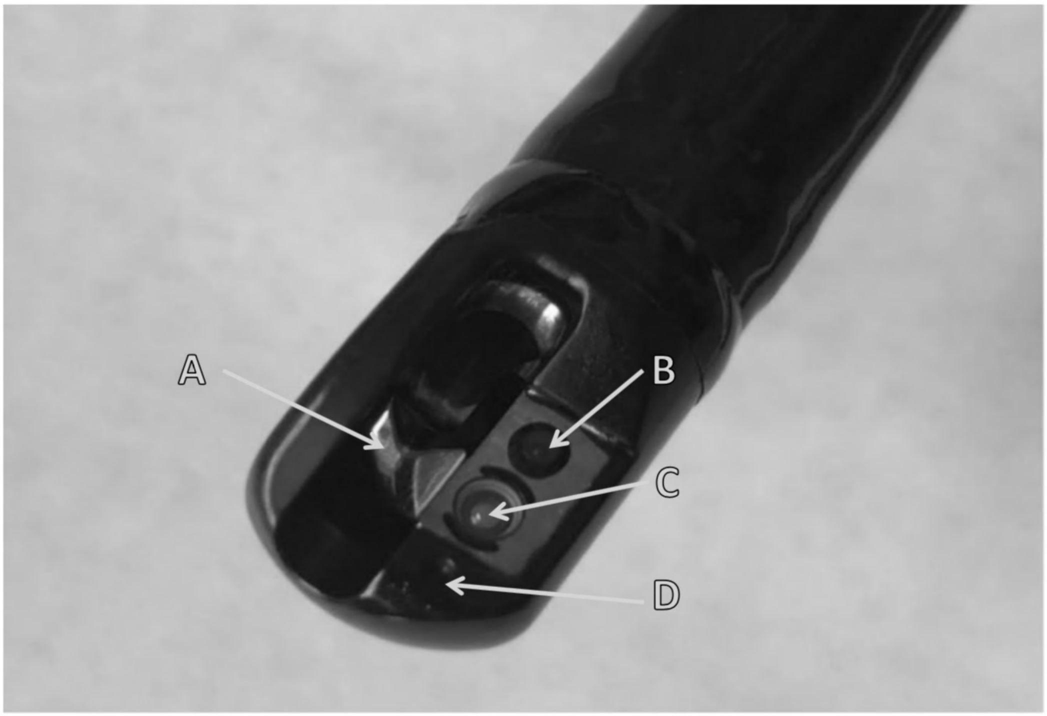


**Figure 1. Duodenoscope tip. A: elevator recess and mechanism; B: fiberoptic camera; C: light source; D: air and water channel. From Rahman *et al.* Dig Dis Sci 2019.[6]**

Due to their specific design, duodenoscopes are among the most complicated medical devices that require routine disinfection and reprocessing. This is mainly a result of the largely inaccessible elevator recess, which makes manual or automated cleansing and disinfection difficult, and therefore promotes persistent pathogenic colonization of the duodenoscope. As a result, there has always been concern over the potential for duodenoscopes to harbor and transmit pathogens between patients. Until relatively recently, this concern has been primarily theoretical. Infection transmission via endoscopy in general is extremely low, estimated to occur at a rate of less than 1 in 1 million procedures.[7] Up until the early 1990s, endoscopy-related infections, while occurring rarely, had always been associated with breaches in published cleaning, disinfecting or drying protocols, or use of unapproved or defective devices or disinfectants.[8]

More recently, however, there has been a sharp rise in the rate of duodenoscope-related infections. A 2016 report identified no less than 25 distinct outbreaks caused by contaminated duodenoscopes worldwide between 2012 and 2015 alone, affecting 250 patients,[9] with at least a dozen of these having arisen from the United States.[10,11] Alarmingly, a large proportion of these

outbreaks have been unrelated to any identifiable breaches in disinfection and reprocessing protocols, and there has been no apparent association with geographic location, duodenoscope manufacturer or model.[9] This has called into question the robustness of traditional sterilization processes as well as traditional duodenoscope designs.

More worrisome is the fact that duodenoscope-related infections and outbreaks are increasingly involving multidrug-resistant organisms (MDROs), which portend significantly higher rates of patient morbidity and mortality compared with traditional organisms. Among the most feared of these are the carbapenem-resistant Enterobacteriaceae (CRE), a family of gram-negative bacteria that has been repeatedly linked to duodenoscope-related infection transmission, and one that is resistant to almost all classes of available antibiotics.[12] [11,13] In order for a duodenoscope-related outbreak to occur, an index patient colonized with CRE or other pathogenic bacteria must undergo an ERCP procedure. Following this, the duodenoscope becomes colonized with the pathogen despite standard sterilization, and another patient who undergoes ERCP with the same duodenoscope then becomes colonized and/or infected.[14] A 2018 nationwide European study demonstrated that nearly 40% of sites providing ERCP had one or more patient-ready duodenoscope(s) that were contaminated after standard processing,[15] suggesting that our current disinfection protocols are inadequate when faced with this latest generation of MDROs. Pathogenic transmission in gastrointestinal endoscopy appears to be rising sharply in the last decade, with specific increases in the rates of MDRO transmission.[16] The mortality rates associated with duodenoscope-related MDRO outbreaks can be devastating, with a 1-month mortality rate of over 20% reported in one American study.[17]

Thus, it is clear that novel strategies need to be developed in order to prevent future ERCP-related outbreaks. Existing sterilization and processing protocols are inadequate, given the increased resistance of the microbial profile colonizing duodenoscopes, particularly behind the inaccessible elevator recess. Multiple manufacturers are therefore attempting to alter the design of traditional duodenoscopes in attempts to address this vital issue. Disposable duodenoscopes are being developed to circumvent the sterilization process, but the cost associated with such devices likely makes them unfeasible for use at medium- or high-volume centers.[18] Specialized duodenoscopes with disposable caps have been developed, and are now approved for use in Canada. The elevator mechanism is housed entirely within the disposable cap, eliminating the primary mechanism behind persistent duodenoscope colonization and patient transmission. Adding an estimated additional ERCP procedural cost of only 3%, these devices are a promising

new modality that have the potential to drastically reduce the rates of duodenoscope-related infection if widely adopted. However, their clinical efficacy and residual contamination rates remain unproven.

## 2. Study Objectives

There are two co-primary objectives of this study; the first is to determine the clinical efficacy of a novel duodenoscope with a disposable elevator cap (DEC™) system compared with a duodenoscope with a traditional design. The second is to determine the rate of persistent pathogenic contamination of the novel duodenoscope following standardized disinfection and reprocessing protocols, compared with a traditional duodenoscope.

## 3. Device Description

This study will be conducted using commercially available devices. All devices will be used in accordance with the appropriate Directions for Use (DFU).

### *3.1. Pentax ED34-i10T2 Duodenoscope*

The Pentax ED34-i10T2 duodenoscope is specifically designed for use with the DEC™ disposable elevator cap. It offers high-definition endoscopic visualization and ergonomic handling while affording the opportunity for complete removal of the duodenoscope cap (which houses the elevator mechanism).

### *3.2. Disposable Elevator Cap (DEC™)*

The DEC™ disposable elevator cap houses the entire elevator mechanism. It is designed for single patient use, being completely sterile, removable and disposable. Removal of the DEC™ allows for manual and automated cleansing of the duodenoscope shaft, controls and tip without concern over persistent microbial colonization of the elevator recess. Of note, we have already informally trialled the ED34-i10T2 with DEC at our center for over 50 cases, with no issues.

### *3.1. Pentax ED34-i10T Duodenoscope*

The Pentax ED34-i10T model is a high-definition duodenoscope currently used in Calgary. Though it has a detachable tip for ease of manual distal end access, the elevator mechanism remains a part of the scope, and the tip is non-disposable, needing to be reattached to the rest of the scope after cleaning.

## 4. Study Design and Setting

This is a prospective, consecutive, randomized controlled study which will assess 1) the clinical efficacy of DEC versus standard duodenoscopes and 2) the persistent bacterial contamination rate of DEC versus standard duodenoscopes. The intervention arm will comprise use of the ED34-i10T2 duodenoscope with DEC, along with any required standard ERCP accessories, whereas the control arm will comprise use of the ED34-i10T duodenoscope with any required ERCP accessories. The study will take place at a single high-volume tertiary ERCP referral center in Calgary, Alberta, Canada, where over 1,500 ERCPs are performed annually. All procedures will be performed by trained endoscopists having each performed over 1,000 ERCPs or by advanced therapeutic endoscopy trainees under direct supervision of the expert endoscopists.

## 5. Study Population

### *5.1. Subject Selection*

All consecutive patients at the study center who meet the eligibility criteria below will be invited for participation in the study. Patients who sign the informed consent form will be considered enrolled. In cases where it is determined that the patient failed to meet the eligibility criteria after the patient has agreed to participate in the study and/or has signed the informed consent, the study personnel will complete screening forms as well as indicate the specific inclusion/exclusion criterion that was not met. Such a patient will be considered a screen failure and will not count towards the final analyses.

### *5.2. Inclusion Criteria*

Patients will be required to meet *all* of the following inclusion criteria in order to be eligible for study participation:

- age $\geq$ 18 years;
- ability to give informed consent to involvement;
- requirement for ERCP to be performed for any indication.

### *5.3. Exclusion Criteria*

Patients meeting *any* of the following exclusion criteria will not be eligible for study participation:

- age < 18 years;
- inability or unwillingness to provide informed consent;
- standard contraindications to ERCP;
- pregnant status or breastfeeding mother;
- inability to successfully complete an ERCP procedure under conscious sedation;
- out-of-province status;
- incarceration.

### *5.4. Randomization and Allocation*

Patients fulfilling the inclusion/exclusion criteria above will be randomized following consent in a 1:1 ratio. Permuted block randomization will be utilized in blocks of 8 patients. Allocation will be to one of two groups: 1) ERCP using ED34-i10T2 duodenoscope with DEC or 2) ERCP using a conventional duodenoscope (ED34-i10T). Confidential random number allocation will be accessed via a secure internet software. The study investigators, outcome adjudicators and patients will be blinded to allocation. The endoscopist will not be blinded. In cases where additional or alternate duodenoscope(s) is/are required to achieve the desired goal of the ERCP procedure, the choice(s) of device(s) will be left to the discretion of the endoscopist, regardless of the study arm to which the patient has been allocated.

## 6. Data Management

### *6.1. Data Sources*

The investigators have previously designed and implemented the Calgary Registry for Advanced and Therapeutic Endoscopy (CReATE, University of Calgary Conjoint Health Research Ethics Board 18-0410). CReATE is a high-fidelity prospective database that seeks to answer questions in ERCP outcomes research. As such, over 400 data fields are collected in real-time for each ERCP procedure, including detailed patient-, endoscopist- and procedure-based variables. Therefore, CReATE serves as the ideal data collection platform for this randomized trial.

Data are inputted by a full-time research assistant, and stored and managed on a secure web application specializing in healthcare database implementation and maintenance, REDCap.[19] This web portal allows for facile access to secure data. The web portal is secured by the latest security technology. Study participants will be identifiable on the secure platform for purposes of patient contact, follow-up and potential withdrawal. For the purposes of this randomized study, an additional tab will be created in the electronic interface that will allow for entry of the arm to which the patient has been randomized. Whenever data are exported for analysis, they will be de-identified and assigned unique study codes only. The primary investigator will be the custodian of the identifiable electronic data, which will be stored only on a secure server accessible only via combination of password protection and temporary electronic fob.

### *6.2. Study Variables*

As part of CReATE, several patient-, endoscopist- and procedure-related variables are routinely collected. These will all be available to analyze as part of this proposed study. Variables include:

- patient-related:
    - age
    - sex
    - comorbidities
    - surgical history
    - social history
- endoscopist-related:
    - experience and procedural volume of endoscopist
    - presence and stage of trainees
    - degree of procedural involvement of trainees

- procedure-related:
    - indication for ERCP
    - history of prior ERCP(s)
    - luminal and biliary anatomy
    - details of procedure (appearance of papilla, intended duct(s) of interest, cannulation attempts and time, findings/appearance of duct(s), pathology)
    - instruments and devices used
    - stent(s) placed
    - procedural success
    - cannulation time
    - procedure time
    - intraprocedural adverse event(s)
    - sedating medications used
- post-procedural:
    - patient pain and satisfaction scores
    - plan for repeat procedure(s)
    - early and late adverse events within 30 days
    - unplanned presentation to acute care facility or readmission within 30 days.

## 7. Study Outcomes

### *7.1. Primary Outcomes*

There are two co-primary outcomes for this study. The first primary outcome of the study is the **ERCP technical success rate** with the ED34-i10T2 duodenoscope and DEC, compared to the currently available duodenoscope (ED34-i10T). Technical success will be determined in duplicate by two blinded outcome adjudicators with no knowledge of the patient's allocation in the study. The adjudicators will review redacted and de-identified procedure reports for each study patient, and determine the presence or absence of technical success of the overall procedure based on a set of *a priori* definitions (see below). Any potential disagreements will be resolved by a third blinded adjudicator. Technical success will also be determined subjectively on a case-by-case basis by the endoscopist performing the procedure, in order to measure correlation with the adjudicator determinations, but not to be used in the determination of the co-primary outcome.

Failure of the procedure due to inability to safely perform the procedure under conscious sedation will not constitute a technical failure for purposes of this study, in either study arm.

- The second co-primary outcome is the **persistent bacterial contamination rate** with the ED34-i10T2 duodenoscope and DEC, compared to the currently available duodenoscope (ED34-i10T) following standardized disinfection and reprocessing. any growth of ≥ 20 colony-forming units (CFUs) at 72 hours, and/or
- any growth of one or more Gram-negative pathogen regardless of CFU count.

### *7.1.1 Definitions of Technical Success*

Technical success will be dependent on the indication for the ERCP, as shown in Table 1. These definitions will be used by the blinded adjudicators to determine the presence or absence of technical success on a case-by-case basis.

Table 1. Definitions of Technical Success by ERCP Indication.

| Indication for ERCP | Definition(s) of Technical Success |
|---|---|
| Suspected or confirmed bile duct stone(s) | Extraction of stone(s)<br><br>OR<br><br>CBD clearance based on absence of filling defects on occlusion cholangiogram<br><br>*If difficult biliary stones are encountered during procedure, use 'Difficult biliary stone(s)' indication below, and above definition does not apply |
| Difficult biliary stone(s)[20-22] – any of:<br>• One or more stone(s) ≥ 15 mm<br>• Barrel or other unusual shape<br>• Multiple (4 or more stones)<br>• Impacted stone(s)<br>• Intrahepatic or cystic duct stone(s)<br>• Stricture below stone(s) | Extraction of stone(s)<br><br>OR<br><br>CBD clearance based on absence of filling defects on occlusion cholangiogram<br><br>OR<br><br>Stenting of CBD as part of future plan to clear duct |
| Biliary stricture (benign or malignant) | Successful placement of stent with proximal margin proximal to stricture<br><br>OR<br><br>Successful dilatation of stricture |
| Cholangioscopy or pancreatoscopy | Successful cholangioscopic or pancreatoscopic visualization of area of interest |

| | |
|---|---|
| Chronic pancreatitis, pancreatic stone(s) and/or pancreatic stricture(s) | Successful cannulation of main pancreatic duct (PD)<br><br>AND AT LEAST 1 OF:<br><br>Pancreatic sphincterotomy<br>Stenting or dilatation of PD<br>Extraction of PD stone(s) |
| Pancreas divisum | Successful minor papilla cannulation<br><br>AND<br><br>Successful pancreatic sphincterotomy |
| Stent removal or exchange | Successful removal and/or exchange of stent(s) |
| Treatment of peri-ampullary bleeding | Successful endoscopic hemostasis |
| Sphincter of Oddi dysfunction | Successful biliary sphincterotomy |

Furthermore, the reason(s) for technical failure will be documented by the adjudicators as per the procedural report (Table 2).

Table 2. Reasons for Technical Failure by ERCP Indication.

| **Reason(s) for Technical Failure** |
|---|
| Inability to locate papilla in patient with normal anatomy<br><br>Inability to locate papilla in patient with altered anatomy, including:<br>• duodenal or peri-ampullary diverticulum<br>• Billroth II surgery<br>• Roux-en-Y surgery<br><br>Inability to achieve an en-face view of the papilla of interest due to a luminal stricture<br><br>Inability to cannulate duct of interest<br><br>Inability to perform sphincterotomy when necessary<br><br>Inability to clear duct of interest<br><br>Inability to place stent proximal to area of interest<br><br>Inability to remove or exchange stent<br><br>Inability to achieve hemostasis endoscopically<br><br>Inability to successfully load, exchange or remove devices necessary for the completion of the procedure<br>Inability to safely complete procedure due to issues with sedation* |

*If this is the only reason for technical failure, the patient will be excluded from final analysis

### *7.2. Secondary Outcomes*

The following will be recorded as secondary outcomes for the study:

- clinical success rate, defined as technical success rate in addition to a lack of repeat unplanned endoscopy, imaging, emergency department presentation, admission within 30 days of the index procedure for reasons related to ongoing pancreaticobiliary pathology that was initially thought to be resolved after the initial ERCP;
- subjective presence or absence of technical success as deemed by the endoscopist;
- assessment of overall ease of use of duodenoscope, using Likert scale of 1-10;
- procedure time;
- device failure or dislodgement rate of the disposable cap;
- overall adverse event rate (intraprocedural, early and late) and severity as defined and graded by the American Society for Gastrointestinal Endoscopy (ASGE) Lexicon[23] within 30 days of the index procedure;
- pancreatitis rate, as defined by new or distinct abdominal pain after ERCP in addition to lipase rise above 3 times the upper limit of normal within 30 days of the index procedure;
- bleeding rate, as defined by hematemesis and/or melena and/or hematochezia, or drop in hemoglobin by $\geq$ 2 g[23] following ERCP with either sphincterotomy or sphincteroplasty (or both) within 30 days of the index procedure;
- cholangitis and/or sepsis rate within 30 days of the index procedure;
- hospital admission or unplanned presentation to an acute healthcare facility within 30 days of the index procedure;
- mortality rate within 30 days of the index procedure;
- manual post-ERCP disinfection time (in endoscopy unit);
- reprocessing time (in automated reprocessing room).

## 8. Study Period and Sequences

### *8.1. Index Procedure*

Consecutive patients undergoing ERCP will be flagged as potentially eligible for study participation. On the day of the procedure, the patient will be approached by a research assistant (RA), who will explain the study, confirm eligibility, and answer any potential questions. If the patient agrees to participate, the relevant informed consent form is signed and witnessed. If the patient decides not to participate, the ERCP proceeds as per the usual standard of care, with or without the use of DEC as per the endoscopist's discretion (and the sequence that follows does not apply).

1. The RA collects any relevant patient-related and pre-procedural data through combination of direct patient interview and review of the medical records.
2. The patient is randomized to one of the two study arms.
3. The patient enters the endoscopy/fluoroscopy room and the procedure commences.
4. During the ERCP procedure, relevant peri-procedural data are recorded by the RA by direct observation, and/or, if necessary, in consultation with the procedural physician(s) and/or nurse(s).
5. After the procedure is complete, the patient is moved to the recovery room for observation. Depending on their disposition and the course of the procedure, observation times and protocols differ. During this stage, the RA collects any relevant post-procedural data, and any missing pre- or peri-procedural data.
6. Any peri- or immediate post-procedural adverse event(s) is/are recorded.
7. The patient is examined by the endoscopist prior to discharge and provided with written post-procedure information, including a phone number to call in the event of any issues.
8. The RA meets with the patient prior to their discharge from the unit to answer any final potential questions.

### *8.2. Cleaning and Reprocessing*

After the ERCP is complete, the in-room nursing team will carry out standardized post-endoscopy manual cleaning and disinfection of the DEC or traditional duodenoscope. In cases where the DEC was used, the cap will be disposed of. These processes will be timed by the research assistant. Following this, the scope will be portered to the automated reprocessing room for disinfection and reprocessing, which will also be timed.

### *8.3. Sampling*

After reprocessing, staff will then observe the following sequence to determine whether there is any persistent bacterial contamination despite standardized disinfection and reprocessing. These samples will then be shipped for microbiologic analysis. The protocol for duodenoscope sampling that will be utilized for this study is adapted from the Duodenoscope Surveillance Sampling and Culturing Protocols, which were developed jointly by the Department of Health and Human Services, Food and Drug Administration (FDA), Centers for Disease Control and Prevention (CDC), and American Society for Microbiology (ASM).[24] The procedure is summarized here.

1. Two staff are needed to conduct sampling from duodenoscope channels; one person (the sampler) will maintain aseptic handling and conduct brushing steps, while the second person (the facilitator) will open packages and handle the unsampled portions of the duodenoscope.
2. The staff will label the sterile sample containers with relevant information.
3. The staff will don their personal protective equipment (PPE).
4. The staff will set the duodenoscope on a sterile drape.
5. Two samples will be collected and combined. First, an instrument channel sample (from the biopsy port to the distal scope end) will be taken.
6. Second, an elevator recess sample will be taken by flushing and brushing of the elevator recess (in the case of the disposable cap, the distal scope tip to which the disposable cap attaches will be sampled instead, ensuring sampling of the joint line between cap and scope).
7. The duodenoscope will then be handled and transported back to the endoscopy unit according to our local institutional policies.
8. Samples will be packed in appropriate biohazard containers, packaged, and transported for microbiologic analysis. The samples will be assigned a unique study number so that the microbiologic analysts will be blinded to the type of duodenoscope that was used for the index procedure.

### *8.4. Microbiologic Analysis*

The presence of any post-reprocessing bacterial population(s) will be assessed using plating for growth in aerobic conditions. If after 72 hours there is no growth, the sample will be deemed negative, and will be discarded. Positive samples will further be characterized using

serial dilutions to establish colony-forming unit (CFU) counts and sequenced for characterization of bacterial communities using a 16S rRNA amplicon Next-generation sequencing (NGS).

***8.5. Follow-up***

Patients will be contacted by the RA by telephone 30 days following their ERCP to assess for any ongoing symptoms and advise of any adverse events, including unplanned emergency department visits or inpatient admissions. The patient's medical record is also reviewed at 30 days for complete details of any unplanned emergency department visits, inpatient admissions or prolonged admissions.

***8.5. Participant Withdrawal***

In accordance with the Declaration of Helsinki and the International Conference on Harmonisation of Technical Requirements for Registration of Pharmaceuticals for Human Use Good Practice Guidelines, a participant is free to withdraw from participation in the study at any time, for any reason without prejudice to their future medical care by the physician or the institution. The investigator(s) may also withdraw the participant at any time in the interests of patient safety. Participants may be removed from the study if one or more of the following events occur:

- withdrawal of consent;
- decision is made by the investigator(s) that removal from the study is in the patient's best medical interest;
- study is stopped by ethics/regulatory authorities.

If a subject withdraws from the clinical investigation, the primary or any additional reason(s) shall be reported. All applicable case report forms up to the point of subject withdrawal, including an End of Study form, should be completed.

Subjects who are "lost-to-follow-up" will have three documented attempts to contact them prior to completion of the End of Study form. If withdrawal is due to investigator's discretion, the investigator should describe what follow-up activities the investigator shall perform. Unless the withdrawal is due to a Serious Adverse Event (SAE), additional subject data will not be collected after the point at which the subject has been withdrawn or withdraws consent from the study. Data collected up to the point of withdrawal may be used by the investigators as permitted in the Informed Consent Form (ICF).

### *8.6. End-of-Study Definition*

This clinical trial will be considered complete when participants are no longer being examined or the last participant's final 30-day follow-up check has occurred. A participant will be considered to have completed the study if he or she has completed the index procedure in addition to the follow-up phone assessment at the 30-day timepoint.

## 9. Statistical Considerations

### *9.1. Hypothesis and Sample Size Calculations*

The first co-primary outcome considers technical success of the ERCP procedure. Overall technical success rates in ERCP vary widely depending on the definitions employed. It is widely agreed upon that a cannulation rate greater than 90% (in native papillae) is an attainable key performance indicator (KPI) in ERCP performed for choledocholithiasis.[25-29] However, in cases of malignant biliary obstruction, especially at low-volume centers, cannulation rates appear to be significantly lower.[30] When one considers overall procedural success rate, these targets become even less clear, with studies reporting between 60 and 90% success rates depending on the definitions employed.[26,30]

Prospective data from our center have been analyzed to assess our group's rates, based on 10 months of procedures in over 600 patients having undergone ERCP. Deep cannulation rates in native papillae at our center are approximately 96%. Overall procedural success in all-comers is approximately 92%, with 4% representing cannulation failures, 4% representing inability to clear or relieve obstruction in the duct(s) of interest.

Thus, when comparing clinical efficacy between DEC and traditional duodenoscopes, we selected an overall baseline technical success rate of 92% based on our experience. We propose that technical success of DEC within a margin of 7% of traditional duodenoscopes will result in an ability to conclude non-inferiority of DEC. A hypothesis will therefore be tested that the overall technical success rate with DEC will be at least 85%:

$$H_0: P_{CON} \geq P_{DEC} + d \text{ (Inferior)} \qquad H_1: P_{CON} - d < P_{DEC} \text{ (Non-inferior)}$$

where $P_{DEC}$ is the overall technical success rate for DEC in this study, $P_{CON}$ is the overall technical success rate for traditionally designed duodenoscopes (control), d is the set non-inferiority limit of 7%, and $H_0$ and $H_1$ are the null and alternate hypotheses, respectively.

An exact test will be used to test the one-sided hypothesis that DEC is non-inferior to control duodenoscopes. Using a power of 80% with a one-sided alpha of 0.025,[31] 236 subjects in each arm will be required, for a total of 472 patients. To compensate for possible loss of subjects due to attrition, an additional 10% of subjects will be enrolled, for a **total of 520 subjects**. It is therefore estimated that the study will require **9-12 months to complete**, based on the estimated volume of ERCPs and study enrolment rates at our center. No interim analyses will be planned.

The second co-primary outcome considers persistent bacterial contamination rate of the duodenoscopes after ERCP. A recent nation-wide analysis showed a persistent bacterial contamination rate of 22% after standard disinfection and reprocessing of duodenoscopes.[15] This was common across multiple duodenoscope manufacturers and models. In particular, the Pentax ED34-i10T duodenoscope demonstrated a persistent contamination rate of 27%, though there was a small sample size of 11 assessing this group.[15] No other available studies have reported specifically on reprocessing of ED34-i10T.

Thus, when comparing persistent contamination between DEC and traditional duodenoscopes, we set an overall expected persistent contamination rate of 20% in the control arm. We propose that reduction in persistent bacterial contamination from 20% with ED34-i10T to 5% with DEC (a relative risk reduction of 75%) would be considered clinically significant. A hypothesis will therefore be tested that the persistent bacterial colonization rate with DEC will be 5% or lower:

$$H_0: P_{DEC} \geq P_{CON} \text{ (Equivalent or Inferior)} \qquad H_1: P_{DEC} < P_{CON} \text{ (Superior)}$$

where $P_{DEC}$ is the persistent contamination rate for DEC in this study, $P_{CON}$ is the persistent contamination rate for traditionally designed duodenoscopes (control), and $H_0$ and $H_1$ are the null and alternate hypotheses, respectively.

An exact test will be used to test the two-sided hypothesis that DEC is superior to control duodenoscopes in terms of persistent bacterial contamination. Using a power of 80% with a two-sided alpha of 0.05, 88 subjects in each arm will be required, for a total of 176 patients. To compensate for possible errors in sampling or loss of samples through transportation, an additional 10% of duodenoscopes will be sampled, for a **total of 194 subjects**. For the purposes of the second co-primary objective of the study, the first 194 patients enrolled as part of the first co-primary objective will have their duodenoscopes sampled (97 from each arm).

## 11. References


1. Maple JT, Ikenberry SO, Anderson MA, et al. The role of endoscopy in the management of choledocholithiasis. *Gastrointestinal endoscopy.* 2011;74(4):731-744.
2. Adler DG, Baron TH, Davila RE, et al. ASGE guideline: the role of ERCP in diseases of the biliary tract and the pancreas. *Gastrointestinal endoscopy.* 2005;62(1):1-8.
3. Anderson MA, Appalaneni V, Ben-Menachem T, et al. The role of endoscopy in the evaluation and treatment of patients with biliary neoplasia. *Gastrointestinal endoscopy.* 2013;77(2):167-174.
4. Chandrasekhara V, Chathadi KV, Acosta RD, et al. The role of endoscopy in benign pancreatic disease. *Gastrointestinal endoscopy.* 2015;82(2):203-214.
5. Chandrasekhara V, Khashab MA, Muthusamy VR, et al. Adverse events associated with ERCP. *Gastrointestinal endoscopy.* 2017;85(1):32-47.
6. Rahman MR, Perisetti A, Coman R, Bansal P, Chhabra R, Goyal H. Duodenoscope-Associated Infections: Update on an Emerging Problem. *Digestive diseases and sciences.* 2019;64(6):1409-1418.
7. Nelson DB, Barkun AN, Block KP, et al. Technology status evaluation report. Transmission of infection by gastrointestinal endoscopy. May 2001. *Gastrointestinal endoscopy.* 2001;54(6):824-828.
8. Spach D, Silverstein F, Stamm W. Transmission of infection by gastrointestinal endoscopy and bronchoscopy. *Annals of internal medicine.* 1993;118:117–128.
9. Preventable tragedies: Superbugs and How Ineffective Monitoring of Medical Device Safety Fails Patients In: U.S. Senate Health E, Labor, and Pensions Committee, ed2016.
10. Smith Z, Young S, Saeian K. Transmission of carbapenem-resistant Enterobacteriaciae during ERCP: time to revisit the current reprocessing guidelines. *Gastrointestinal endoscopy.* 2015;81:1041-1045.
11. Epstein L, Hunter J, Arwady M. New Delhi metallo-beta-lactamase-producing carbapenem-resistant Escherichia coli associated with exposure to duodenoscopes. *Jama.* 2014;312:1447-1455.
12. Verfaillie C, Bruno M, Holt A. Withdrawal of a novel-design duodenoscope ends outbreak of a VIM-2-producing Pseudomonas aeruginosa. *Endoscopy.* 2015;47:502.
13. Aumeran C, Poincloux L, Souweine B. Multidrug-resistant Klebsiella pneumoniae outbreak after endoscopic retrograde cholangiopancreatography. *Endoscopy.* 2010;42:895-899.
14. Kim S, Russell D, Mohamadnejad M, et al. Risk factors associated with the transmission of carbapenem-resistant Enterobacteriaceae via contaminated duodenoscopes. *Gastrointestinal endoscopy.* 2016;83(6):1121-1129.
15. Rauwers AW, Voor In 't Holt AF, Buijs JG, et al. High prevalence rate of digestive tract bacteria in duodenoscopes: a nationwide study. *Gut.* 2018;67(9):1637-1645.
16. Kovaleva J, Peters FT, van der Mei HC, Degener JE. Transmission of infection by flexible gastrointestinal endoscopy and bronchoscopy. *Clinical microbiology reviews.* 2013;26(2):231-254.
17. Ross AS, Baliga C, Verma P, Duchin J, Gluck M. A quarantine process for the resolution of duodenoscope-associated transmission of multidrug-resistant Escherichia coli. *Gastrointestinal endoscopy.* 2015;82(3):477-483.
18. Bang JY, Sutton B, Hawes R, Varadarajulu S. Concept of disposable duodenoscope: at what cost? *Gut.* 2019.

19. Harris P, Taylor R, Thielke R, Payne J, Gonzalez N, Conde J. Research electronic data capture (REDCap) – A metadata-driven methodology and workflow process for providing translational research informatics support. *J Biomed Inform.* 2009;42(2):377-381.
20. Brewer Gutierrez OI, Bekkali NLH, Raijman I, et al. Efficacy and Safety of Digital Single-Operator Cholangioscopy for Difficult Biliary Stones. *Clinical gastroenterology and hepatology : the official clinical practice journal of the American Gastroenterological Association.* 2018;16(6):918-926.e911.
21. Stefanidis G, Christodoulou C, Manolakopoulos S, Chuttani R. Endoscopic extraction of large common bile duct stones: A review article. *World journal of gastrointestinal endoscopy.* 2012;4(5):167-179.
22. McHenry L, Lehman G. Difficult bile duct stones. *Curr Treat Options Gastroenterol.* 2006;9(2):123-132.
23. Cotton PB, Eisen GM, Aabakken L, et al. A lexicon for endoscopic adverse events: report of an ASGE workshop. *Gastrointestinal endoscopy.* 2010;71(3):446-454.
24. Duodenoscope Surveillance Sampling and Culturing Protocols. In: Services DoHaH, ed2018.
25. Domagk D, Oppong KW, Aabakken L, et al. Performance measures for ERCP and endoscopic ultrasound: a European Society of Gastrointestinal Endoscopy (ESGE) Quality Improvement Initiative. *Endoscopy.* 2018;50(11):1116-1127.
26. Wadsworth CA, Dwyer LK, Paranandi B, et al. Current performance of ERCP in the clearance of bile duct stones in UK centres-working towards robust key performance indicators. *Gastrointestinal endoscopy.* 2014;Conference:Digestive Diease Week, DDW 2014 ASGE. Chicago, IL United States. Conference Publication: (var.pagings). 2079 (2015 SUPPL. 2011) (pp AB2230).
27. Chathadi KV, Chandrasekhara V, Acosta RD, et al. The role of ERCP in benign diseases of the biliary tract. *Gastrointestinal endoscopy.* 2015;81(4):795-803.
28. Enochsson L, Swahn F, Arnelo U, Nilsson M, Lohr M, Persson G. Nationwide, population-based data from 11,074 ERCP procedures from the Swedish Registry for Gallstone Surgery and ERCP. *Gastrointestinal endoscopy.* 2010;72(6):1175-1184, 1184.e1171-1173.
29. Cretu I, Kelleher B, Stewart S, Bennett G, MacMathuna P, Leyden J. Audit of ERCP key performance indicators in a high volume interventional/biliary endoscopy centre. *Irish Journal of Medical Science.* 2015;Conference:Irish Society of Gastroentrology, Summer Meeting 2014. Naas Ireland. Conference Publication: (var.pagings). 2184 (2013 SUPPL. 2011) (pp S2099).
30. Donnan E, Bentrem DJ, Komanduri S, Mahvi DM, Keswani RN. ERCP in potentially resectable malignant biliary obstruction is frequently unsuccessful when performed outside of a comprehensive pancreaticobiliary center. *Journal of surgical oncology.* 2016;113(6):647-651.
31. Julious SA, Campbell MJ. Tutorial in biostatistics: sample sizes for parallel group clinical trials with binary data. *Statistics in medicine.* 2012;31(24):2904-2936.

**STUDY PROTOCOL**

# Clinical Efficacy and Infection Risk of ERCP using a Novel Duodenoscope with a Disposable Cap: a Randomized Controlled Trial

Short Title: Infection Control in ERCP using a Duodenoscope with a Disposable Cap (ICECAP)


Principal Investigator: Nauzer Forbes MD MSc[1]
Clinical Assistant Professor
Division of Gastroenterology
Cumming School of Medicine, University of Calgary
TRW 6D19, 3280 Hospital Dr. NW
T2N 4Z6 Calgary, AB
p 403.592.5089 f 403.592.5090
nauzer.forbes@ucalgary.ca

Co-investigators: B Joseph Elmunzer MD MSc[2]
Steven J Heitman MD MSc[1]
Andre G Buret PhD[1]
Thibault Allain PhD[1]
Sydney Bass MD[1]
Paul Belletrutti MD[1]
Martin J Cole MD[1]
Emmanuel Gonzalez-Moreno MD[1]
Ahmed Kayal MD[1]
Puja Kumar MD[1]
Rachid Mohamed MD[1]
Christian Turbide MD[1]

Affiliations: [1]University of Calgary, Calgary, Alberta, Canada
[2]Medical University of South Carolina, Charleston, South Carolina, USA



Supported by: The American Society for Gastrointestinal Endoscopy (ASGE)


Protocol Version: 8.0

## 1. Introduction

### *1.1. Background*

Endoscopic retrograde cholangiopancreatography (ERCP) is well recognized as an important therapeutic modality for biliary and/or pancreatic pathology. There are various established roles of ERCP, including: the first-line modality for management of choledocholithiasis,[1] the first-line modality for decompressive management of pancreatico-biliary strictures,[2] the first-line modality (in conjunction with cholangioscopy) for evaluation and/or treatment of proximal biliary neoplasia,[3] and an important modality in the treatment of several benign pancreatico-biliary disorders, including Sphincter of Oddi dysfunction and pancreas divisum.[4] In conjunction with other endoscopic, surgical and radiographic techniques, ERCP is therefore a cornerstone in the management of pancreatico-biliary disease. While effective, ERCP has several well-established associated adverse events. These include post-ERCP pancreatitis (PEP), bleeding, cholangitis, cholecystitis, perforation, and cardiopulmonary events.[5]

ERCP is universally performed using a highly specialized type of flexible endoscope called a duodenoscope. Rather than having a forward-facing camera at the end of the scope (which is typical of gastroscopes and colonoscopes), duodenoscopes have a side-viewing camera close to the distal tip that faces perpendicularly to the long axis of the scope. A representation of a typical duodenoscope tip is shown in Figure 1. The unique design of a duodenoscope affords direct visualization of the major and/or minor papillae, which is a requisite for successful biliary and/or pancreatic cannulation and instrumentation. In addition, a recess in the tip of the duodenoscope houses an elevator, a device designed to assist with fine tip control of pancreatico-biliary cannulation and extraction devices.

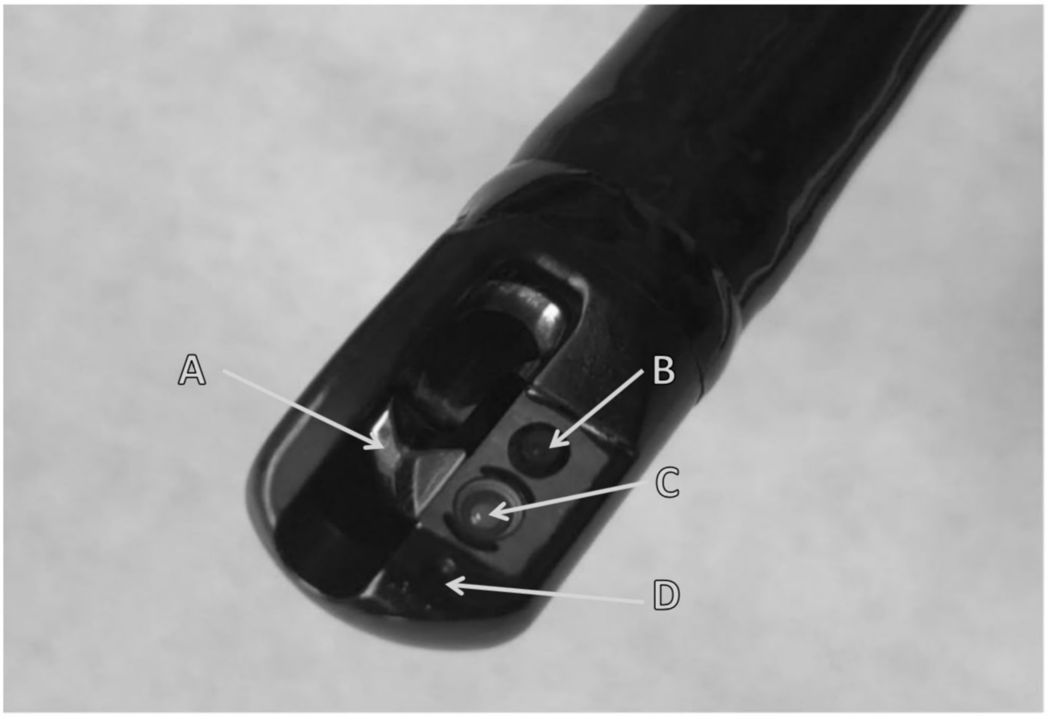


**Figure 1. Duodenoscope tip. A: elevator recess and mechanism; B: fiberoptic camera; C: light source; D: air and water channel. From Rahman *et al.* Dig Dis Sci 2019.[6]**

Due to their specific design, duodenoscopes are among the most complicated medical devices that require routine disinfection and reprocessing. This is mainly a result of the largely inaccessible elevator recess, which makes manual or automated cleansing and disinfection difficult, and therefore promotes persistent pathogenic colonization of the duodenoscope. As a result, there has always been concern over the potential for duodenoscopes to harbor and transmit pathogens between patients. Until relatively recently, this concern has been primarily theoretical. Infection transmission via endoscopy in general is extremely low, estimated to occur at a rate of less than 1 in 1 million procedures.[7] Up until the early 1990s, endoscopy-related infections, while occurring rarely, had always been associated with breaches in published cleaning, disinfecting or drying protocols, or use of unapproved or defective devices or disinfectants.[8]

More recently, however, there has been a sharp rise in the rate of duodenoscope-related infections. A 2016 report identified no less than 25 distinct outbreaks caused by contaminated duodenoscopes worldwide between 2012 and 2015 alone, affecting 250 patients,[9] with at least a dozen of these having arisen from the United States.[10,11] Alarmingly, a large proportion of these

outbreaks have been unrelated to any identifiable breaches in disinfection and reprocessing protocols, and there has been no apparent association with geographic location, duodenoscope manufacturer or model.[9] This has called into question the robustness of traditional sterilization processes as well as traditional duodenoscope designs.

More worrisome is the fact that duodenoscope-related infections and outbreaks are increasingly involving multidrug-resistant organisms (MDROs), which portend significantly higher rates of patient morbidity and mortality compared with traditional organisms. Among the most feared of these are the carbapenem-resistant Enterobacteriaceae (CRE), a family of gram-negative bacteria that has been repeatedly linked to duodenoscope-related infection transmission, and one that is resistant to almost all classes of available antibiotics.[12] [11,13] In order for a duodenoscope-related outbreak to occur, an index patient colonized with CRE or other pathogenic bacteria must undergo an ERCP procedure. Following this, the duodenoscope becomes colonized with the pathogen despite standard sterilization, and another patient who undergoes ERCP with the same duodenoscope then becomes colonized and/or infected.[14] A 2018 nationwide European study demonstrated that nearly 40% of sites providing ERCP had one or more patient-ready duodenoscope(s) that were contaminated after standard processing,[15] suggesting that our current disinfection protocols are inadequate when faced with this latest generation of MDROs. Pathogenic transmission in gastrointestinal endoscopy appears to be rising sharply in the last decade, with specific increases in the rates of MDRO transmission.[16] The mortality rates associated with duodenoscope-related MDRO outbreaks can be devastating, with a 1-month mortality rate of over 20% reported in one American study.[17]

Thus, it is clear that novel strategies need to be developed in order to prevent future ERCP-related outbreaks. Existing sterilization and processing protocols are inadequate, given the increased resistance of the microbial profile colonizing duodenoscopes, particularly behind the inaccessible elevator recess. Multiple manufacturers are therefore attempting to alter the design of traditional duodenoscopes in attempts to address this vital issue. Disposable duodenoscopes are being developed to circumvent the sterilization process, but the cost associated with such devices likely makes them unfeasible for use at medium- or high-volume centers.[18] Specialized duodenoscopes with disposable caps have been developed, and are now approved for use in Canada. The elevator mechanism is housed entirely within the disposable cap, eliminating the primary mechanism behind persistent duodenoscope colonization and patient transmission. Adding an estimated additional ERCP procedural cost of only 3%, these devices are a promising

new modality that have the potential to drastically reduce the rates of duodenoscope-related infection if widely adopted. However, their clinical efficacy and residual contamination rates remain unproven.

## 2. Study Objectives

There are two co-primary objectives of this study; the first is to determine the clinical efficacy of a novel duodenoscope with a disposable elevator cap (DEC™) system compared with a duodenoscope with a traditional design. The second is to determine the rate of persistent pathogenic contamination of the novel duodenoscope following standardized disinfection and reprocessing protocols, compared with a traditional duodenoscope.

## 3. Device Description

This study will be conducted using commercially available devices. All devices will be used in accordance with the appropriate Directions for Use (DFU).

### *3.1. Pentax ED34-i10T2 Duodenoscope*

The Pentax ED34-i10T2 duodenoscope is specifically designed for use with the DEC™ disposable elevator cap. It offers high-definition endoscopic visualization and ergonomic handling while affording the opportunity for complete removal of the duodenoscope cap (which houses the elevator mechanism).

### *3.2. Disposable Elevator Cap (DEC™)*

The DEC™ disposable elevator cap houses the entire elevator mechanism. It is designed for single patient use, being completely sterile, removable and disposable. Removal of the DEC™ allows for manual and automated cleansing of the duodenoscope shaft, controls and tip without concern over persistent microbial colonization of the elevator recess. Of note, we have already informally trialled the ED34-i10T2 with DEC at our center for over 50 cases, with no issues.

### *3.1. Pentax ED34-i10T Duodenoscope*

The Pentax ED34-i10T model is a high-definition duodenoscope currently used in Calgary. Though it has a detachable tip for ease of manual distal end access, the elevator mechanism

remains a part of the scope, and the tip is non-disposable, needing to be reattached to the rest of the scope after cleaning.

**4. Study Design and Settings**

This is a prospective, consecutive, randomized controlled study which will assess 1) the clinical efficacy of DEC versus standard duodenoscopes and 2) the persistent bacterial contamination rate of DEC versus standard duodenoscopes. The intervention arm will comprise use of the ED34-i10T2 duodenoscope with DEC, along with any required standard ERCP accessories, whereas the control arm will comprise use of the ED34-i10T duodenoscope with any required ERCP accessories. The study will take place at a two high-volume tertiary ERCP referral centers: 1) the Peter Lougheed Centre in Calgary, Alberta, Canada, where over 1,600 ERCPs are performed annually and 2) Kingston General Hospital in Kingston, Ontario, Canada where over 600 ERCPs are performed annually. All procedures will be performed by trained endoscopists having each performed over 1,000 ERCPs or by advanced therapeutic endoscopy trainees under direct supervision of the expert endoscopists.

**5. Study Population**

***5.1. Subject Selection***

All consecutive patients at the study center who meet the eligibility criteria below will be invited for participation in the study. Patients who sign the informed consent form will be considered enrolled. In cases where it is determined that the patient failed to meet the eligibility criteria after the patient has agreed to participate in the study and/or has signed the informed consent, the study personnel will complete screening forms as well as indicate the specific inclusion/exclusion criterion that was not met. Such a patient will be considered a screen failure and will not count towards the final analyses.

***5.2. Inclusion Criteria***

Patients will be required to meet *all* of the following inclusion criteria in order to be eligible for study participation:

- age $\geq$ 18 years;
- ability to give informed consent to involvement;

- requirement for ERCP to be performed for any indication.

### *5.3. Exclusion Criteria*

Patients meeting *any* of the following exclusion criteria will not be eligible for study participation:

- age < 18 years;
- inability or unwillingness to provide informed consent;
- standard contraindications to ERCP;
- pregnant status or breastfeeding mother;
- inability to successfully complete an ERCP procedure under conscious sedation;
- out-of-province status;
- incarceration.

### *5.4. Randomization and Allocation*

Patients fulfilling the inclusion/exclusion criteria above will be randomized following consent in a 1:1 ratio. Permuted block randomization will be utilized in blocks of 8 patients. Allocation will be to one of two groups: 1) ERCP using ED34-i10T2 duodenoscope with DEC or 2) ERCP using a conventional duodenoscope (ED34-i10T). Confidential random number allocation will be accessed via a secure internet software. The study investigators, outcome adjudicators and patients will be blinded to allocation. The endoscopist will not be blinded. In cases where additional or alternate duodenoscope(s) is/are required to achieve the desired goal of the ERCP procedure, the choice(s) of device(s) will be left to the discretion of the endoscopist, regardless of the study arm to which the patient has been allocated.

## 6. Data Management

### *6.1. Data Sources*

The investigators have previously designed and implemented the Calgary Registry for Advanced and Therapeutic Endoscopy (CReATE, University of Calgary Conjoint Health Research Ethics Board 18-0410). CReATE is a high-fidelity prospective database that seeks to answer questions in ERCP outcomes research. As such, over 400 data fields are collected in real-time for each ERCP procedure, including detailed patient-, endoscopist- and procedure-based

variables. Therefore, CReATE serves as the ideal data collection platform for this randomized trial.

Data are inputted by a full-time research assistant, and stored and managed on a secure web application specializing in healthcare database implementation and maintenance, REDCap.[19] This web portal allows for facile access to secure data. The web portal is secured by the latest security technology. Study participants will be identifiable on the secure platform for purposes of patient contact, follow-up and potential withdrawal. For the purposes of this randomized study, an additional tab will be created in the electronic interface that will allow for entry of the arm to which the patient has been randomized. Whenever data are exported for analysis, they will be de-identified and assigned unique study codes only. The primary investigator will be the custodian of the identifiable electronic data, which will be stored only on a secure server accessible only via combination of password protection and temporary electronic fob.

### *6.2. Study Variables*

As part of CReATE, several patient-, endoscopist- and procedure-related variables are routinely collected. These will all be available to analyze as part of this proposed study. Variables include:

- patient-related:
    - age
    - sex
    - comorbidities
    - surgical history
    - social history
- endoscopist-related:
    - experience and procedural volume of endoscopist
    - presence and stage of trainees
    - degree of procedural involvement of trainees
- procedure-related:
    - indication for ERCP
    - history of prior ERCP(s)
    - luminal and biliary anatomy

    - details of procedure (appearance of papilla, intended duct(s) of interest, cannulation attempts and time, findings/appearance of duct(s), pathology)
    - instruments and devices used
    - stent(s) placed
    - procedural success
    - cannulation time
    - procedure time
    - intraprocedural adverse event(s)
    - sedating medications used
- post-procedural:
    - patient pain and satisfaction scores
    - early and late adverse events within 30 days
    - unplanned presentation to acute care facility or readmission within 30 days, with details.

## 7. Study Outcomes

### *7.1. Primary Outcomes*

There are two co-primary outcomes for this study. The first primary outcome of the study is the **ERCP technical success rate** with the ED34-i10T2 duodenoscope and DEC, compared to the currently available duodenoscope (ED34-i10T). Technical success will be determined in duplicate by two blinded outcome adjudicators with no knowledge of the patient's allocation in the study. The adjudicators will review redacted and de-identified procedure reports for each study patient, and determine the presence or absence of technical success of the overall procedure based on a set of *a priori* definitions (see below). Any potential disagreements will be resolved by a third blinded adjudicator. Technical success will also be determined subjectively on a case-by-case basis by the endoscopist performing the procedure, in order to measure correlation with the adjudicator determinations, but not to be used in the determination of the co-primary outcome. Failure of the procedure due to inability to safely perform the procedure under conscious sedation will not constitute a technical failure for purposes of this study, in either study arm. The second co-primary outcome is the **persistent bacterial contamination rate** with the ED34-i10T2

duodenoscope and DEC, compared to the currently available duodenoscope (ED34-i10T) following standardized disinfection and reprocessing. This will be defined as:

- any growth of ≥ 10 colony-forming units (CFUs) at 72 hours, and/or
- any growth of one or more Gram-negative pathogen regardless of CFU count.

### *7.1.1 Definitions of Technical Success*

Technical success will be dependent on the indication for the ERCP, as shown in Table 1. These definitions will be used by the blinded adjudicators to determine the presence or absence of technical success on a case-by-case basis.

Table 1. Definitions of Technical Success by ERCP Indication.

| Indication for ERCP | Definition(s) of Technical Success |
|---|---|
| Suspected or confirmed bile duct stone(s) | Extraction of stone(s)<br><br>OR<br><br>CBD clearance based on absence of filling defects on occlusion cholangiogram<br><br>*If difficult biliary stones are encountered during procedure, use 'Difficult biliary stone(s)' indication below, and above definition does not apply |
| Difficult biliary stone(s)[20-22] – any of:<br>• One or more stone(s) ≥ 15 mm<br>• Barrel or other unusual shape<br>• Multiple (4 or more stones)<br>• Impacted stone(s)<br>• Intrahepatic or cystic duct stone(s)<br>• Stricture below stone(s) | Extraction of stone(s)<br><br>OR<br><br>CBD clearance based on absence of filling defects on occlusion cholangiogram<br><br>OR<br><br>Stenting of CBD as part of future plan to clear duct |
| Biliary stricture (benign or malignant) | Successful placement of stent with proximal margin proximal to stricture<br><br>OR<br><br>Successful dilatation of stricture |
| Cholangioscopy or pancreatoscopy | Successful cholangioscopic or pancreatoscopic visualization of area of interest |
| Chronic pancreatitis, pancreatic stone(s) and/or pancreatic stricture(s) | Successful cannulation of main pancreatic duct (PD)<br><br>AND AT LEAST 1 OF:<br><br>Pancreatic sphincterotomy<br>Stenting or dilatation of PD<br>Extraction of PD stone(s) |
| Pancreas divisum | Successful minor papilla cannulation |

| | |
|---|---|
| | AND<br><br>Successful pancreatic sphincterotomy |
| Stent removal or exchange | Successful removal and/or exchange of stent(s) |
| Treatment of peri-ampullary bleeding | Successful endoscopic hemostasis |
| Sphincter of Oddi dysfunction | Successful biliary sphincterotomy |

Furthermore, the reason(s) for technical failure will be documented by the adjudicators as per the procedural report (Table 2).

Table 2. Reasons for Technical Failure by ERCP Indication.

| **Reason(s) for Technical Failure** |
|---|
| Inability to locate papilla in patient with normal anatomy<br><br>Inability to locate papilla in patient with altered anatomy, including:<br>• duodenal or peri-ampullary diverticulum<br>• Billroth II surgery<br>• Roux-en-Y surgery<br><br>Inability to achieve an en-face view of the papilla of interest due to a luminal stricture<br><br>Inability to cannulate duct of interest<br><br>Inability to perform sphincterotomy when necessary<br><br>Inability to clear duct of interest<br><br>Inability to place stent proximal to area of interest<br><br>Inability to remove or exchange stent<br><br>Inability to achieve hemostasis endoscopically<br><br>Inability to successfully load, exchange or remove devices necessary for the completion of the procedure<br>Inability to safely complete procedure due to issues with sedation*<br><br>*If this is the only reason for technical failure, the patient will be excluded from final analysis |

### *7.2. Secondary Outcomes*

The following will be recorded as secondary outcomes for the study:

- clinical success rate, defined as technical success rate in addition to a lack of repeat unplanned endoscopy, imaging, emergency department presentation, admission within 30 days of the index procedure for reasons related to ongoing pancreaticobiliary pathology that was initially thought to be resolved after the initial ERCP;
- subjective presence or absence of technical success as deemed by the endoscopist;
- assessment of overall ease of use of duodenoscope, using Likert scale of 1-10;
- procedure time;
- device failure or dislodgement rate of the disposable cap;
- overall adverse event rate (intraprocedural, early and late) and severity as defined and graded by the American Society for Gastrointestinal Endoscopy (ASGE) Lexicon[23] within 30 days of the index procedure;
- pancreatitis rate, as defined by new or distinct abdominal pain after ERCP in addition to lipase rise above 3 times the upper limit of normal within 30 days of the index procedure;
- bleeding rate, as defined by hematemesis and/or melena and/or hematochezia, or drop in hemoglobin by $\geq$ 2 g[23] following ERCP with either sphincterotomy or sphincteroplasty (or both) within 30 days of the index procedure;
- cholangitis and/or sepsis rate within 30 days of the index procedure;
- hospital admission or unplanned presentation to an acute healthcare facility within 30 days of the index procedure;
- mortality rate within 30 days of the index procedure;
- manual post-ERCP disinfection time (in endoscopy unit);
- reprocessing time (in automated reprocessing room).

## 8. Study Period and Sequences

### *8.1. Index Procedure*

Consecutive patients undergoing ERCP will be flagged as potentially eligible for study participation. On the day of the procedure, the patient will be approached by a research assistant (RA), who will explain the study, confirm eligibility, and answer any potential questions. If the patient agrees to participate, the relevant informed consent form is signed and witnessed. If the patient decides not to participate, the ERCP proceeds as per the usual standard of care, with or

without the use of DEC as per the endoscopist's discretion (and the sequence that follows does not apply).

1. The RA collects any relevant patient-related and pre-procedural data through combination of direct patient interview and review of the medical records.
2. The patient is randomized to one of the two study arms.
3. The patient enters the endoscopy/fluoroscopy room and the procedure commences.
4. During the ERCP procedure, relevant peri-procedural data are recorded by the RA by direct observation, and/or, if necessary, in consultation with the procedural physician(s) and/or nurse(s).
5. After the procedure is complete, the patient is moved to the recovery room for observation. Depending on their disposition and the course of the procedure, observation times and protocols differ. During this stage, the RA collects any relevant post-procedural data, and any missing pre- or peri-procedural data.
6. Any peri- or immediate post-procedural adverse event(s) is/are recorded.
7. The patient is examined by the endoscopist prior to discharge and provided with written post-procedure information, including a phone number to call in the event of any issues.
8. The RA meets with the patient prior to their discharge from the unit to answer any final potential questions.

### *8.2. Cleaning and Reprocessing*

After the ERCP is complete, the in-room nursing team will carry out standardized post-endoscopy manual cleaning and disinfection of the DEC or traditional duodenoscope. In cases where the DEC was used, the cap will be disposed of. These processes will be timed by the research assistant. Following this, the scope will be portered to the automated reprocessing room for disinfection and reprocessing, which will also be timed.

### *8.3. Sampling*

After reprocessing, staff will then observe the following sequence to determine whether there is any persistent bacterial contamination despite standardized disinfection and reprocessing. These samples will then be shipped for microbiologic analysis. The protocol for duodenoscope sampling that will be utilized for this study is adapted from the Duodenoscope Surveillance Sampling and Culturing Protocols, which were developed jointly by the Department

of Health and Human Services, Food and Drug Administration (FDA), Centers for Disease Control and Prevention (CDC), and American Society for Microbiology (ASM).[24] The procedure is summarized here.

1. Two staff are needed to conduct sampling from duodenoscope channels; one person (the sampler) will maintain aseptic handling and conduct brushing steps, while the second person (the facilitator) will open packages and handle the unsampled portions of the duodenoscope.
2. The staff will label the sterile sample containers with relevant information.
3. The staff will don their personal protective equipment (PPE).
4. The staff will set the duodenoscope on a sterile drape.
5. Two samples will be collected and combined. First, an instrument channel sample (from the biopsy port to the distal scope end) will be taken.
6. Second, an elevator recess sample will be taken by flushing and brushing of the elevator recess (in the case of the disposable cap, the distal scope tip to which the disposable cap attaches will be sampled instead, ensuring sampling of the joint line between cap and scope).
7. The duodenoscope will then be handled and transported back to the endoscopy unit according to our local institutional policies.
8. Samples will be packed in appropriate biohazard containers, packaged, and transported for microbiologic analysis. The samples will be assigned a unique study number so that the microbiologic analysts will be blinded to the type of duodenoscope that was used for the index procedure.

### *8.4. Microbiologic Analysis*

The presence of any microbial population(s) will be assessed using plating for growth in aerobic conditions. 0.1 mL of wash fluid will be plated to blood agar, MacConkey agar, and Columbia blood agar with colistin-nalidixic acid. Growth will be reported in colony-forming units (CFU)/mL. If after 72 hours there is no growth, the sample will be deemed negative, and will be discarded. Positive samples will be further characterized. For Gram-negative bacilli (GNB), these will be reported with CFU/mL and the isolated organism(s) will be reported as identified by matrix-

assisted laser desorption/ionization (MALDI). Non-GNB organisms will be reported with CFU/mL and a group identification (ie, coagulase-negative *Staphylococcus*, or *Candida*).

### *8.5. Follow-up*

Patients will be contacted by the RA by telephone 30 days following their ERCP to assess for any ongoing symptoms and advise of any adverse events, including unplanned emergency department visits or inpatient admissions. The patient's medical record is also reviewed at 30 days for complete details of any unplanned emergency department visits, inpatient admissions or prolonged admissions.

### *8.5. Participant Withdrawal*

In accordance with the Declaration of Helsinki and the International Conference on Harmonisation of Technical Requirements for Registration of Pharmaceuticals for Human Use Good Practice Guidelines, a participant is free to withdraw from participation in the study at any time, for any reason without prejudice to their future medical care by the physician or the institution. The investigator(s) may also withdraw the participant at any time in the interests of patient safety. Participants may be removed from the study if one or more of the following events occur:

- withdrawal of consent;
- decision is made by the investigator(s) that removal from the study is in the patient's best medical interest;
- study is stopped by ethics/regulatory authorities.

If a subject withdraws from the clinical investigation, the primary or any additional reason(s) shall be reported. All applicable case report forms up to the point of subject withdrawal, including an End of Study form, should be completed.

Subjects who are "lost-to-follow-up" will have three documented attempts to contact them prior to completion of the End of Study form. If withdrawal is due to investigator's discretion, the investigator should describe what follow-up activities the investigator shall perform. Unless the withdrawal is due to a Serious Adverse Event (SAE), additional subject data will not be collected after the point at which the subject has been withdrawn or withdraws consent from the study. Data collected up to the point of withdrawal may be used by the investigators as permitted in the Informed Consent Form (ICF).

### *8.6. End-of-Study Definition*

This clinical trial will be considered complete when participants are no longer being examined or the last participant's final 30-day follow-up check has occurred. A participant will be considered to have completed the study if he or she has completed the index procedure in addition to the follow-up phone assessment at the 30-day timepoint.

## 9. Statistical Considerations

### *9.1. Hypothesis and Sample Size Calculations*

The first co-primary outcome considers technical success of the ERCP procedure. Overall technical success rates in ERCP vary widely depending on the definitions employed. It is widely agreed upon that a cannulation rate greater than 90% (in native papillae) is an attainable key performance indicator (KPI) in ERCP performed for choledocholithiasis.[25-29] However, in cases of malignant biliary obstruction, especially at low-volume centers, cannulation rates appear to be significantly lower.[30] When one considers overall procedural success rate, these targets become even less clear, with studies reporting between 60 and 90% success rates depending on the definitions employed.[26,30]

Prospective data from our center have been analyzed to assess our group's rates, based on 10 months of procedures in over 600 patients having undergone ERCP. Deep cannulation rates in native papillae at our center are approximately 96%. Overall procedural success in all-comers is approximately 92%, with 4% representing cannulation failures, 4% representing inability to clear or relieve obstruction in the duct(s) of interest.

Thus, when comparing clinical efficacy between DEC and traditional duodenoscopes, we selected an overall baseline technical success rate of 92% based on our experience. We propose that technical success of DEC within a margin of 7% of traditional duodenoscopes will result in an ability to conclude non-inferiority of DEC. A hypothesis will therefore be tested that the overall technical success rate with DEC will be at least 85%:

$$H_0: P_{CON} \geq P_{DEC} + d \text{ (Inferior)} \qquad H_1: P_{CON} - d < P_{DEC} \text{ (Non-inferior)}$$

where $P_{DEC}$ is the overall technical success rate for DEC in this study, $P_{CON}$ is the overall technical success rate for traditionally designed duodenoscopes (control), d is the set non-inferiority limit of 7%, and $H_0$ and $H_1$ are the null and alternate hypotheses, respectively.

An exact test will be used to test the one-sided hypothesis that DEC is non-inferior to control duodenoscopes. Using a power of 80% with a one-sided alpha of 0.025,[31] 236 subjects in each arm will be required, for a total of 472 patients. To compensate for possible loss of subjects due to attrition, an additional 10% of subjects will be enrolled, for a **total of 520 subjects**. It is therefore estimated that the study will require **9-12 months to complete**, based on the estimated volume of ERCPs and study enrolment rates at our center. No interim analyses will be planned.

The second co-primary outcome considers persistent bacterial contamination rate of the duodenoscopes after ERCP. A recent nation-wide analysis showed a persistent bacterial contamination rate of 22% after standard disinfection and reprocessing of duodenoscopes.[15] This was common across multiple duodenoscope manufacturers and models. In particular, the Pentax ED34-i10T duodenoscope demonstrated a persistent contamination rate of 27%, though there was a small sample size of 11 assessing this group.[15] No other published studies have reported specifically on reprocessing of ED34-i10T. However, real-world data from the FDA demonstrate a 9.3% total sample positivity rate among Pentax duodenoscopes.[32] Furthermore, our preliminary data based on post-reprocessing bioluminescence scans demonstrate that 10% of ED34-i10T duodenoscopes require repeat re-processing due to counts representative of residual contamination.

Thus, when comparing persistent contamination between DEC and traditional duodenoscopes, we set an overall expected persistent contamination rate of 10% in the control arm. We propose that reduction in persistent bacterial contamination from 10% with ED34-i10T to 3% with DEC (a relative risk reduction of 67%) would be considered clinically significant. A hypothesis will therefore be tested that the persistent bacterial colonization rate with DEC will be 3% or lower:

$$H_0: P_{DEC} \geq P_{CON} \text{ (Equivalent or Inferior)} \qquad H_1: P_{DEC} < P_{CON} \text{ (Superior)}$$

where $P_{DEC}$ is the persistent contamination rate for DEC in this study, $P_{CON}$ is the persistent contamination rate for traditionally designed duodenoscopes (control), and $H_0$ and $H_1$ are the null and alternate hypotheses, respectively.

An exact test will be used to test the two-sided hypothesis that DEC is superior to control duodenoscopes in terms of persistent bacterial contamination. Using a power of 80% with a two-sided alpha of 0.05, 194 subjects in each arm will be required, for a total of 388 patients. To compensate for possible errors in sampling or loss of samples through transportation, an additional 10% of duodenoscopes will be sampled, for a **total of 426 subjects**. For the purposes

of the second co-primary objective of the study, the first 426 patients enrolled as part of the first co-primary objective will have their duodenoscopes sampled (213 from each arm). An anticipated 25% of the sample size will be recruited from Kingston.

## 11. References


1. Maple JT, Ikenberry SO, Anderson MA, et al. The role of endoscopy in the management of choledocholithiasis. *Gastrointestinal endoscopy.* 2011;74(4):731-744.
2. Adler DG, Baron TH, Davila RE, et al. ASGE guideline: the role of ERCP in diseases of the biliary tract and the pancreas. *Gastrointestinal endoscopy.* 2005;62(1):1-8.
3. Anderson MA, Appalaneni V, Ben-Menachem T, et al. The role of endoscopy in the evaluation and treatment of patients with biliary neoplasia. *Gastrointestinal endoscopy.* 2013;77(2):167-174.
4. Chandrasekhara V, Chathadi KV, Acosta RD, et al. The role of endoscopy in benign pancreatic disease. *Gastrointestinal endoscopy.* 2015;82(2):203-214.
5. Chandrasekhara V, Khashab MA, Muthusamy VR, et al. Adverse events associated with ERCP. *Gastrointestinal endoscopy.* 2017;85(1):32-47.
6. Rahman MR, Perisetti A, Coman R, Bansal P, Chhabra R, Goyal H. Duodenoscope-Associated Infections: Update on an Emerging Problem. *Digestive diseases and sciences.* 2019;64(6):1409-1418.
7. Nelson DB, Barkun AN, Block KP, et al. Technology status evaluation report. Transmission of infection by gastrointestinal endoscopy. May 2001. *Gastrointestinal endoscopy.* 2001;54(6):824-828.
8. Spach D, Silverstein F, Stamm W. Transmission of infection by gastrointestinal endoscopy and bronchoscopy. *Annals of internal medicine.* 1993;118:117–128.
9. Preventable tragedies: Superbugs and How Ineffective Monitoring of Medical Device Safety Fails Patients In: U.S. Senate Health E, Labor, and Pensions Committee, ed2016.
10. Smith Z, Young S, Saeian K. Transmission of carbapenem-resistant Enterobacteriaciae during ERCP: time to revisit the current reprocessing guidelines. *Gastrointestinal endoscopy.* 2015;81:1041-1045.
11. Epstein L, Hunter J, Arwady M. New Delhi metallo-beta-lactamase-producing carbapenem-resistant Escherichia coli associated with exposure to duodenoscopes. *Jama.* 2014;312:1447-1455.
12. Verfaillie C, Bruno M, Holt A. Withdrawal of a novel-design duodenoscope ends outbreak of a VIM-2-producing Pseudomonas aeruginosa. *Endoscopy.* 2015;47:502.
13. Aumeran C, Poincloux L, Souweine B. Multidrug-resistant Klebsiella pneumoniae outbreak after endoscopic retrograde cholangiopancreatography. *Endoscopy.* 2010;42:895-899.
14. Kim S, Russell D, Mohamadnejad M, et al. Risk factors associated with the transmission of carbapenem-resistant Enterobacteriaceae via contaminated duodenoscopes. *Gastrointestinal endoscopy.* 2016;83(6):1121-1129.
15. Rauwers AW, Voor In 't Holt AF, Buijs JG, et al. High prevalence rate of digestive tract bacteria in duodenoscopes: a nationwide study. *Gut.* 2018;67(9):1637-1645.

16. Kovaleva J, Peters FT, van der Mei HC, Degener JE. Transmission of infection by flexible gastrointestinal endoscopy and bronchoscopy. *Clinical microbiology reviews.* 2013;26(2):231-254.
17. Ross AS, Baliga C, Verma P, Duchin J, Gluck M. A quarantine process for the resolution of duodenoscope-associated transmission of multidrug-resistant Escherichia coli. *Gastrointestinal endoscopy.* 2015;82(3):477-483.
18. Bang JY, Sutton B, Hawes R, Varadarajulu S. Concept of disposable duodenoscope: at what cost? *Gut.* 2019.
19. Harris P, Taylor R, Thielke R, Payne J, Gonzalez N, Conde J. Research electronic data capture (REDCap) – A metadata-driven methodology and workflow process for providing translational research informatics support. *J Biomed Inform.* 2009;42(2):377-381.
20. Brewer Gutierrez OI, Bekkali NLH, Raijman I, et al. Efficacy and Safety of Digital Single-Operator Cholangioscopy for Difficult Biliary Stones. *Clinical gastroenterology and hepatology : the official clinical practice journal of the American Gastroenterological Association.* 2018;16(6):918-926.e911.
21. Stefanidis G, Christodoulou C, Manolakopoulos S, Chuttani R. Endoscopic extraction of large common bile duct stones: A review article. *World journal of gastrointestinal endoscopy.* 2012;4(5):167-179.
22. McHenry L, Lehman G. Difficult bile duct stones. *Curr Treat Options Gastroenterol.* 2006;9(2):123-132.
23. Cotton PB, Eisen GM, Aabakken L, et al. A lexicon for endoscopic adverse events: report of an ASGE workshop. *Gastrointestinal endoscopy.* 2010;71(3):446-454.
24. Duodenoscope Surveillance Sampling and Culturing Protocols. In: Services DoHaH, ed2018.
25. Domagk D, Oppong KW, Aabakken L, et al. Performance measures for ERCP and endoscopic ultrasound: a European Society of Gastrointestinal Endoscopy (ESGE) Quality Improvement Initiative. *Endoscopy.* 2018;50(11):1116-1127.
26. Wadsworth CA, Dwyer LK, Paranandi B, et al. Current performance of ERCP in the clearance of bile duct stones in UK centres-working towards robust key performance indicators. *Gastrointestinal endoscopy.* 2014;Conference:Digestive Diease Week, DDW 2014 ASGE. Chicago, IL United States. Conference Publication: (var.pagings). 2079 (2015 SUPPL. 2011) (pp AB2230).
27. Chathadi KV, Chandrasekhara V, Acosta RD, et al. The role of ERCP in benign diseases of the biliary tract. *Gastrointestinal endoscopy.* 2015;81(4):795-803.
28. Enochsson L, Swahn F, Arnelo U, Nilsson M, Lohr M, Persson G. Nationwide, population-based data from 11,074 ERCP procedures from the Swedish Registry for Gallstone Surgery and ERCP. *Gastrointestinal endoscopy.* 2010;72(6):1175-1184, 1184.e1171-1173.
29. Cretu I, Kelleher B, Stewart S, Bennett G, MacMathuna P, Leyden J. Audit of ERCP key performance indicators in a high volume interventional/biliary endoscopy centre. *Irish Journal of Medical Science.* 2015;Conference:Irish Society of Gastroentrology, Summer Meeting 2014. Naas Ireland. Conference Publication: (var.pagings). 2184 (2013 SUPPL. 2011) (pp S2099).
30. Donnan E, Bentrem DJ, Komanduri S, Mahvi DM, Keswani RN. ERCP in potentially resectable malignant biliary obstruction is frequently unsuccessful when performed outside of a comprehensive pancreaticobiliary center. *Journal of surgical oncology.* 2016;113(6):647-651.

31. Julious SA, Campbell MJ. Tutorial in biostatistics: sample sizes for parallel group clinical trials with binary data. *Statistics in medicine.* 2012;31(24):2904-2936.
32. The FDA Provides Interim Results of Duodenoscope Reprocessing Studies Conducted in Real-World Settings: FDA Safety Communication. https://www.fda.gov/medical-devices/safety-communications/fda-provides-interim-results-duodenoscope-reprocessing-studies-conducted-real-world-settings-fda. Published 2018. Accessed.

**Statistical Analysis Plan – ICECAP**
**v1.0 MARCH 16, 2022**

## A) Data groups:

1. MICROBIOLOGY OUTCOME – co-primary outcome 1 (superiority outcome). N = 422 patients.
2. TECHNICAL OUTCOME – co-primary outcome 2 (non-inferiority outcome). N = 518 patients.
3. SAFETY OUTCOMES AND SECONDARY OUTCOMES. N = 518 patients.

## B) Variables and data dictionary:

| Variable Name | Description/ Definition | Coding |
|---|---|---|
| study_id | unique study identifier | (whole number) |
| scope_random | study randomization and allocation | 1 = scope design A; 2 = scope design B (blinded) |
| tech_outcome | technical success, co-primary outcome #1 (non-inferiority) | 0 = unsuccessful; 1 = successful |
| micro_outcome | persistent microbial contamination, co-primary outcome #2 (superiority) | 0 = no event; 1 = event |
| ae_pancreatitis | pancreatitis following ERCP (ascertained at 30-day follow-up) | 0 = no event; 1 = event |
| ae_bleeding | bleeding following ERCP (ascertained at 30-day follow-up) | 0 = no event; 1 = event |
| ae_perforation | perforation following ERCP (ascertained at 30-day follow-up) | 0 = no event; 1 = event |
| ae_cholang | cholangitis following ERCP (ascertained at 30-day follow-up) | 0 = no event; 1 = event |
| ae_cardio_resp | cardiorespiratory adverse event following ERCP (ascertained at 30-day follow-up) | 0 = no event; 1 = event |
| ae_death | death following ERCP (ascertained at 30-day follow-up) | 0 = no event; 1 = event |
| ice_duo_num | duodenoscope number | (one of several pre-specified numbers) |
| ice_duo_ease_md | ease of use of overall scope design, according to endoscopist/trainee | (scale from 0 to 10) |

| | | |
|---|---|---|
| ice_duo_ease_rn | ease of use of overall scope design, according to assisting/cleaning nurse | (scale from 0 to 10) |
| ice_duo_cross | crossover to alternate scope design/ study arm | 0 = no crossover; 1 = crossover |
| age | patient age | (continuous) |
| sex | patient sex | 1 = female; 2 = male |
| charlson | Charlson comorbidity index | (whole number scale from 0 to 33) |
| disposition | patient disposition | 0 = outpatient; 1 = inpatient |
| trainee | trainee participation in ERCP | 0 = no trainee; 1 = trainee present |
| indication | indication for ERCP | 1 = ampullary obstruction or stricture; 2 = bile leak; 3 = benign or anastomotic biliary stricture; 4 = suspected or confirmed malignant biliary obstruction; 5 = cholangitis; 6 = suspected or confirmed biliary stones; 7 = repeat planned procedure including stent removal or exchange; 8 = other |
| complexity | procedural complexity as defined by ASGE grade for complexity of ERCP | 1 = Grade I; 2 = Grade II; 3 = Grade III; 4 = Grade IV |
| ercp_prev | previous ERCP | 0 = no; 1 = yes |
| cbd_or_pd | targetted duct for cannulation | 0 = common bile duct; 1 = pancreatic duct |
| scope_position | scope position during ERCP | 1 = short; 2 = long |
| pre_cut | pre-cut sphincterotomy performed (includes trans-pancreatic or supra-papillary) | 0 = no; 1 = yes |
| nk_papillotomy | needle knife papillotomy performed | 0 = no; 1 = yes |
| cbd_cannulation | CBD cannulation achieved (only calculate among patients where cbd_or_pd = 0) | 0 = no; 1 = yes |
| cannulation_time | cannulation time (only calculate among patients where cbd_cannulation = 1) | (continuous) |
| sphinct | formal sphincterotomy performed with sphincterotome | 0 = no; 1 = yes |
| balloon_sphinct | balloon sphincteroplasty performed | 0 = no; 1 = yes |
| cbd_stones | CBD stones found | 0 = no; 1 = yes |

| | | |
|---|---|---|
| cbd_stricture | CBD stricture found | 0 = no; 1 = yes |
| leak | bile leak found | 0 = no; 1 = yes |
| cholangioscopy | cholangioscopy performed | 0 = no; 1 = yes |
| biliary_stent | biliary stent placed | 0 = no; 1 = yes |
| panc_stent | pancreatic stent placed | 0 = no; 1 = yes |
| proc_time | procedure time (from intubation to extubation) | (continuous) |
| gen_anesth | general anesthesia or monitored anesthesia care performed | 0 = no; 1 = yes |
| midazolam | amount of midazolam given (only calculate for gen_anesth = 0) | 0 = no; 1 = yes |
| fentanyl | amount of midazolam given (only calculate for gen_anesth = 0) | (continuous) |
| glucagon | amount of midazolam given (only calculate for gen_anesth = 0) | (continuous) |
| diphenhydramine | amount of midazolam given (only calculate for gen_anesth = 0) | (continuous) |
| indomethacin | indomethacin administered to mitigate post-ERCP pancreatitis | 0 = no; 1 = yes |
| intra_aware | intra-procedural awareness | (scale from 0 to 10) |
| intra_discomf | intra-procedural discomfort | (scale from 0 to 10) |
| post_pain | post-procedural abdominal pain | (scale from 0 to 10) |
| post_throat | post-procedural throat pain | (scale from 0 to 10) |
| post_nausea | post-procedural nausea | (scale from 0 to 10) |
| post_distend | post-procedural distention | (scale from 0 to 10) |

## C) ANALYSIS

Two-tailed Fisher's exact tests and chi-squared tests will be used to compare proportions. Outcomes will be compared between groups in an intention-to-treat analysis without adjustment. Comparisons will be reported using relative risks (RRs) and numbers needed to treat (NNTs) along with their respective 95% confidence intervals (CIs).

*Post hoc* subgroup analyses will be performed on sex (female or male), age (<65 or ≥65 years), study site (Calgary or Kingston), procedural complexity (1-2 or 3-4), papilla status (native

papilla or prior sphincterotomy), disposition (inpatient or outpatient), biliary stent placement (present or none), and indication (suspected or confirmed malignancy or all others). All statistical analyses will be performed using RStudio version 1.2.1335 (Integrated Development Environment for R, Boston, USA).

**D) OUTPUTS**

1. Table comparing proportions of variables between study groups
2. Summary of RRs, ARRs, RRRs, NNTs, NNHs
3. Subgroup analyses and forest plot of subgroups
