# Supplementary material for: Effect of Disposable Elevator Cap Duodenoscopes on Persistent Microbial Contamination and Technical Performance of Endoscopic Retrograde Cholangiopancreatography: The ICECAP Randomized Clinical Trial
Source: JAMA Intern Med. 2023 Jan 23;183(3):191–200. doi: 10.1001/jamainternmed.2022.6394 (PMC9871945; doi:10.1001/jamainternmed.2022.6394)
Supplement: Supplement 3. — Data Sharing Statement [file jamainternmed-e226394-s003.pdf]

Data Sharing Statement

Forbes. Effect of Disposable Elevator Cap Duodenoscopes on Persistent Microbial Contamination and Technical Performance of Endoscopic Retrograde Cholangiopancreatography. *JAMA Intern Med*. Published January 23, 2023. doi:10.1001/jamainternmed.2022.6394

Data
Data available: Yes
Data types: Other (please specify)
Additional Information: On an independent case-by-case basis upon reasonable request.
How to access data: Requests for data can be sent to nauzer.forbes@ucalgary.ca.
When available: With publication

Supporting Documents
Document types: Statistical/analytic code
How to access documents: These will be provided as an online supplement.
When available: With publication

Additional Information
Who can access the data: On an independent case-by-case basis upon reasonable request.
Types of analyses: On an independent case-by-case basis upon reasonable request.
Mechanisms of data availability: On an independent case-by-case basis upon reasonable request.
